# Category selectivity observed in the human brain is distinct from category selectivity observed in artificial neural networks

**DOI:** 10.64898/2026.05.29.728609

**Published:** 2026-06-02

**Authors:** Alish Dipani, N. Apurva Ratan Murty

**Affiliations:** 1 Center of Excellence in Computational Cognition, Georgia Tech; 2 School of Psychological and Brain Sciences, Georgia Tech

## Abstract

Category selectivity for images of faces, scenes, and bodies is among the most striking and reproducible findings in vision neuroscience. Artificial neural networks (ANNs) trained on visual tasks also develop category-selective units, which has led to the suggestion that ANNs may capture important aspects of how the brain processes visual categories. But the mere presence of category-selective units in ANNs does not mean that those units are selective in the same way as the brain. Here, we distinguish between the presence of category-selective units in ANNs from the form of selectivity they express, and show that the selectivity that emerges in ANN units differs in meaningful and systematic ways from that observed in the human brain with fMRI. To this end, we first identified category-selective units in a wide range of ANN models using standard fMRI localizers, and found that selective units emerged reliably in trained, but not in untrained, ANNs. We then identified category-selective regions in the human brain using the same localizer and found that their response tuning to a broad range of images was strikingly consistent across individuals. Thus, category-selective regions exhibit a stable representational signature shared across subjects. Category-selective ANN units did not match this structure. Their responses diverged in both univariate tuning and multivariate representational geometry, fell well below the human-human ceiling, varied substantially across models, and depended strongly on the localizer used to identify them. We also found that the category-selective ANN units were neither necessary nor sufficient for predicting neural responses using an encoding model. Further stimulus-level analyses revealed clear and interpretable mismatches between ANN selectivity and human fMRI responses, which can be used to test and compare better ANN models in the future. Taken together, these results show that the full range of response tuning in category-selective regions provides a highly demanding and discriminative test of brain-model alignment than previously appreciated. Although current ANNs contain category-selective units, the selectivity they express is more fragile and does not capture the stable and shared form of selectivity observed in the human brain.

## 1 Introduction

Few findings in cognitive neuroscience are as robust or influential as category-selective responses in high-level visual cortex. Regions like the fusiform face area (FFA) [1–3], extrastriate body area (EBA) [4, 5], and parahippocampal place area (PPA) [6–8] respond maximally to faces, bodies, and scenes respectively and have shaped how we think about brain organization, development, and evolution [9–23]. Intriguingly, artificial neural networks (ANNs) trained on a wide variety of tasks also develop category-selective units that respond more strongly to faces, bodies, and scenes than to other categories [24–38]. This parallel has led to the suggestion that ANNs may capture the fundamental computational principles underlying visual category selectivity. But this conclusion assumes that the category-selective units in ANNs reflect the same underlying mechanisms and features found in the brain. In this study, we distinguish between the mere presence of category-selective units in ANNs and the form of category selectivity they exhibit, and show that category selectivity that develops in ANN units is different from the selectivity observed in human category-selective brain regions.

**Figure 1:**
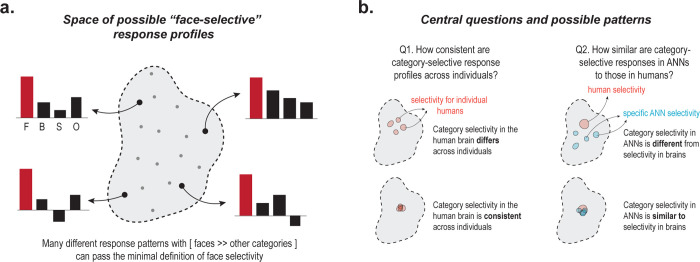
Conceptual framework, questions, and possibility space. **a.** Motivating conceptual framework: The gray blob represents the space of response profiles satisfying the minimal criterion for face selectivity (faces » other categories). Gray and black dots illustrate some sampled face-selective response profiles within the space. Barplots show schematic category-averaged responses for faces (F), bodies (B), scenes (S), and objects (O); faces in red. Many different response profiles can satisfy the minimal definition of face selectivity (faces » other categories). **b.** Schematic illustrating the two central questions explored in this study. Left, possible patterns of category selectivity across humans. Right, possible relationships between category selectivity in humans (red) and ANNs (blue).

The central challenge in interpreting category selectivity is that the standard definition of selectivity is underconstrained. A voxel in the brain (or unit in an ANN model) is typically deemed, say, face-selective if it responds more to faces than to other categories. But as shown in Fig 1a, this minimal criterion admits a wide range of response profiles. For example, both a voxel that responds exclusively to faces and one that responds broadly but slightly more to faces would qualify as face-selective. In fact, there exist *infinitely* many response patterns that can be deemed face-selective (illustrated as the blob in Fig 1a). This flexibility in definition invites a subtle but consequential cognitive bias [39, 40]. Once a voxel or unit is labeled “face-selective”, we are inclined to group such units as conceptually similar to the brain, implicitly assuming that they instantiate a common representational principle. But without carefully examining the full range of the response profile and comparing against the profile observed in brains, we cannot know whether observed selectivity in ANNs is truly brain-like.

This framing leads naturally to two core questions that motivate this study (Fig. 1b). First (Q1), how consistent are category-selective response profiles across individuals? If human selectivity patterns vary substantially across people, then the question of comparing any model to a single canonical pattern would itself be ill-posed. Q1 therefore contrasts two possibilities: either selectivity profiles in human category-selective regions are highly idiosyncratic across people, or they are systematic enough to define a stable population-level reference (Fig 1b, **left**). Second (Q2), do ANN units exhibit the same kind of category-selective response profiles as brain voxels in category-selective regions? Q2 thus contrasts two possibilities: One possibility is that brains and ANN units differ in their selectivity profiles despite both being deemed category-selective by the minimal definition. The second possibility is that ANN-units mirror the structure of category selectivity observed in the brain.

To answer these questions, we leveraged 7T fMRI data from the Natural Scenes Dataset [41], which provides stimulus-level responses to thousands of natural images. Using these data, we asked whether category selectivity is consistent across humans and whether category-selective units in ANNs exhibit the same form of selectivity observed in the brain. To foreshadow our results, we found that the pattern of responses from category-selective regions (FFA, EBA, PPA, and others) was highly consistent across humans. This similarity provided a stable target to compare ANN representations. We then applied a localizer-based procedure (like human fMRI) to ANN models and found that face-, body-, and scene-selective units emerge reliably in trained, but not untrained, ANNs. Do these ANN units exhibit the same *form* of selectivity as human category-selective regions? We found that they do not. Although category-selective ANN units preferred the same category of images, their response patterns were reliably different from those seen in human brains with fMRI. These differences were robust to analysis choices and human-interpretable across models and reveal distinct response signatures in brains and ANNs. Together, these findings reveal a dissociation between the existence of category-selective units and the form of category selectivity they exhibit. This distinction shifts the focus from whether category-selective units emerge to how category selectivity is represented, providing a more quantitative framework for comparing biological and artificial visual systems.

## 2 Results

### 2.1 Category-selective units emerge in trained, but not untrained, ANNs

**Figure 2:**
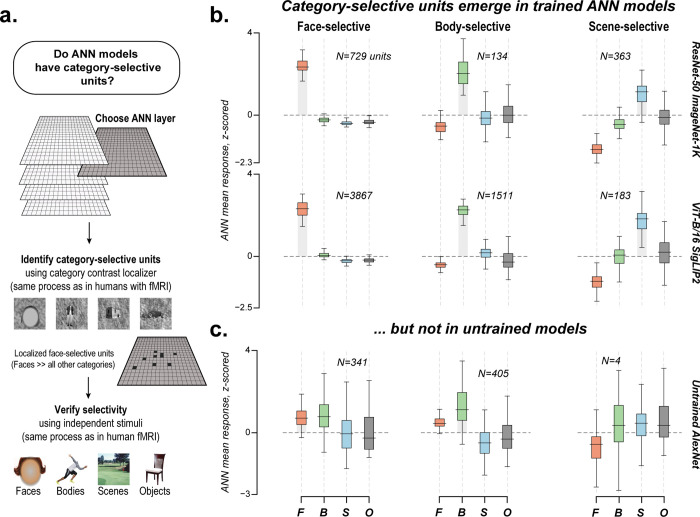
Trained, but not untrained, ANNs have reliable face-, body- and scene-selective units. **a** Schematic of the procedure used to identify category-selective units in a given ANN. For a layer (gray square), we apply a category-localizer [42] to identify candidate category-selective units, and validate their selectivity using an independent set of face, body, scene, and object images [24]. **b** Responses of localized category-selective units (from fROI-corresponding layer, t>7; see Methods) to independent stimuli of faces (F), bodies (B), scenes (S), and objects (O). For all boxplots, the x-axis indicates the stimulus category, and the y-axis shows the unit-averaged z-scored responses. The text indicates the number of category-selective units localized. The first row is for a standard ANN model (ResNet-50 ImageNet-1K) and the second row is for the best-performing model (ViT-B/16 SigLIP2, see Methods). The columns show responses of face-, body-, and scene-selective units, respectively. **c** Same as **b** (except that t-threshold was lowered to t>3, see Methods) but for an untrained AlexNet instance (seed = 5, see Methods).

Do artificial neural networks (ANNs) contain face-, body-, and scene-selective units? For a balanced comparison with brains, we subjected several pretrained ANNs to the same localizer images used to identify category-selective regions in the human brain [42–46]. Specifically, we extracted ANN responses to a widely used fMRI localizer [42] which has also been used in prior studies of category-selectivity in ANNs [24–31, 36]. This dataset includes images of faces, bodies, scenes, objects, characters, and scrambled objects. To identify, say, face-selective units, we looked for units whose response to faces was significantly greater than to every other category at a stringent statistical threshold (t>7, two-sided *p* = 7.17 × 10^−12^, see Methods). Next, we verified the selectivity of the identified units on an independent, publicly available stimulus set containing faces, bodies, scenes, and objects [24]. The procedure is shown schematically in Fig. 2a. The response profiles of the identified category-selective units (in the representative layer, see Methods) for stimulus categories in two representative ANN models (trained ResNet-50 ImageNet-1K and ViT-B/16 SigLIP2) are shown in Fig. 2b, c. The mean response to the preferred category (gray bar) was significantly higher than the response to all non-preferred categories (one-sided Mann-Whitney U tests; all p<0.0001). We repeated the same analysis for 34 other pretrained ANN models (see Methods). These models span a range of architectures, training datasets, learning objectives, and model sizes (see Supplementary Table 1 for the full list). Here too, the response to the preferred category was consistently and significantly higher than for other categories (one-sided Mann-Whitney U tests; all p<0.05, Fig. S1) for all trained model architectures.

Next, we asked if untrained (randomly initialized) ANNs also contain category-selective units. Note that our procedure of identifying category-selective ANN units is quite stringent and requires generalization to an independent stimulus set (as in human fMRI). We evaluated 20 untrained (randomly initialized) model instances (4 architectures × 5 seeds, see Supplementary Table 2 for the full list) using the same procedure. None of the untrained models met the criterion for category selectivity across faces, bodies, and scenes at our originally defined statistical threshold (t>7, Fig. S2). Note that some category-selective units could be detectable at lower selectivity thresholds (t>3, two-sided *p* = 2.82 × 10^−3^). But the selectivity of these units did not consistently generalize to held-out stimuli (see Fig. 2c for an untrained AlexNet instance). These findings are in contrast with some prior claims of selectivity in untrained models [25] and show that model training is in fact essential for category selectivity in ANN units.

Taken together, these results show that category-selective units emerge reliably across a wide range of trained, but not untrained, ANN models. But the mere existence of category-selective ANN units that generalize to held-out images does not guarantee that the *form* of selectivity that arises matches what is observed in the human brain. We tested this next.

### 2.2 Category selectivity in ANNs is distinct from category selectivity in human brains: evidence from univariate measures

**Figure 3:**
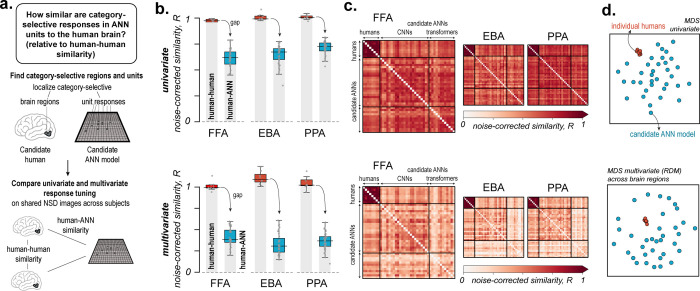
Category selectivity in ANN units is distinct from category selectivity in human brains by univariate and multivariate measures. **a.** Schematic of the analysis. Category-selective regions were identified in human fMRI data, and category-selective units were identified in ANN models using analogous localizer procedures. Responses to shared images from the Natural Scenes Dataset (NSD) were then compared between humans and ANNs using univariate and multivariate measures of similarity. **b.** Noise-corrected similarity between category-selective responses in humans and ANNs. For each region (FFA, EBA, PPA), boxplots compare human-human similarity (left) and human-ANN similarity (right). Each point denotes the median similarity for an individual subject (human-human) or ANN model (human-ANN). Top row, univariate comparisons. Bottom row, multivariate comparisons. Human-human similarity consistently exceeded human-ANN similarity across regions and metrics. **c.** Pairwise similarity matrices for category-selective response profiles. Subjects are arranged in the upper-left quadrant, ANN models in the lower-right quadrant, and human-ANN comparisons occupy the off-diagonal quadrants. Diagonal entries are masked. Darker colors indicate greater noise-corrected similarity. Top row, univariate comparisons. Bottom row, multivariate comparisons. **d.** Multidimensional scaling (MDS) visualization of pairwise similarities, averaged across face-, body-, and scene-selective regions. Each point represents a human subject (red) or ANN model (blue). Top row, univariate comparisons. Bottom row, multivariate comparisons. Human subjects cluster closely together, whereas ANN models occupy a distinct region of representational space.

To meaningfully compare ANN selectivity to the brain, we needed to go beyond broad categories and characterize the full stimulus-level response tuning across a range of images and establish how reproducible those tuning patterns were across individuals (Q1, Fig. 1b). To address this question, we localized the category-selective voxels in the brain and measured the noise-corrected similarity of univariate response tuning across subjects in the FFA, EBA, and PPA using voxel-averaged responses to natural images from the Natural Scenes Dataset (NSD [41], see Methods). For each NSD subject (8 total), we correlated that subject’s voxel-averaged responses with those of each of the remaining seven subjects using a shared set of 515 images. The median of these seven correlations defined the subject-specific inter-subject ceiling. To account for measurement noise, correlations were normalized by the Spearman–Brown corrected within-subject split-half reliability (noise-corrected correlations can slightly exceed 1; see Methods). Responses in the canonical category-selective regions (FFA, EBA, and PPA) were strikingly consistent across individuals, with high noise-corrected human-human correlations (Fig. 3b; median R=0.98 for FFA, 0.99 for EBA, and 1.01 for PPA; all individual brain–brain pair Rs>0.87, p<0.0001). This cross-individual similarity of responses establishes a stringent benchmark for testing the response tuning found in category-selective ANN units (the NeuroAI Turing Test [47]).

#### Do ANN category-selective units exhibit human-like univariate response tuning?

We presented the same shared stimulus set viewed by the NSD subjects to each ANN model and measured the unit-averaged responses of the previously identified face-, body-, and scene-selective units (see S3). Note that the EBA is selective for an entirely different stimulus category (bodies). Thus, face-selective ANN units are less similar to the FFA than the tuning observed between several distinct human category-selective regions. The same pattern held for the EBA and body-selective ANN units (ΔR=0.33) as well as the PPA and scene-selective ANN units (ΔR=0.28), where the model–brain gaps exceeded those observed between different human category-selective regions (S3). Even the best ANN model we considered (ViT-B/16 SigLIP2) was significantly below the human–human ceiling (exact paired sign-flip permutation test across subjects, all one-sided ps<0.05), with substantial gaps of ΔR=0.32 for face selectivity, 0.25 for body selectivity, and 0.31 for scene selectivity. The full similarity pattern across all individual subjects and candidate ANN models sorted by convolutional and transformer architectures is shown in (Fig. 3c). To further visualize these differences across brain regions, we applied multidimensional scaling (MDS; 2 dimensions, see Methods) to the pairwise tuning curves across humans and ANNs (Fig. 3d). Human subjects formed a tight cluster, indicating high similarity with each other. ANN models (blue), on the other hand, were distinct from the human group (and to each other).

Together, these findings show that the voxel-averaged univariate stimulus-level response patterns in category-selective regions like the FFA, EBA, and PPA are remarkably consistent across human brains (in support of the second possibility for Q1, Fig 1b, left), whereas the unit-averaged category-selective responses observed in ANNs diverge significantly from those in humans (in support of the first possibility for Q2, Fig 1b).

### 2.3 Category selectivity in ANNs is distinct from category selectivity in human brains: evidence from multivariate measures

It remains possible that the representations in category-selective ANN units more closely resemble human category-selective representations when considering the multivariate pattern of responses (beyond the voxel/unit-averaged univariate tuning) [48–53]. To directly test this possibility, we compared the multivariate structure between ANNs and brain responses using representational dissimilarity matrices (RDMs) (see Methods). We constructed RDMs based on Euclidean distances between pairs of images across voxels/units (appropriate for categorical stimuli, see [54]). As in the univariate analyses, we accounted for measurement noise by normalizing the pairwise RDM correlations with each subject’s split-half RDM reliability across repeated stimulus presentations. We first asked how consistent the multivariate structure of responses was across human subjects. As for the univariate analyses, we found that RDMs from the FFA, EBA, and PPA were highly similar across participants (Fig. 3b; median R=0.99 for FFA, 1.09 for EBA, and 1.02 for PPA; all individual human-human pair Rs>0.81, p<0.0001). These results show that even the fine-grained multivariate response patterns in category-selective cortex are remarkably stable across individuals (further support possibility 2 for Q1).

#### Do category-selective units in ANNs exhibit multivariate response structure similar to that observed in human category-selective brain regions?

We compared the representational structure of ANN face-, body-, and scene-selective units to that of the FFA, EBA, and PPA in humans. As before, we used the same images to measure noise-corrected correlations between RDMs observed in ANN and in brains. Across all models and category-selective regions, human-ANN correlations were significantly lower than the ceiling established by human-human correlations (S4). Similarly, the median gap (ΔR) between human-human and human-ANN correlations was 0.78 for EBA and 0.68 for PPA. Strikingly, these gaps were larger than those for univariate tests (Fig. 3b). Even for the best-performing model (ViT-B/16 SigLIP2), the human-ANN correlation was significantly below human-human ceiling (exact paired sign-flip permutation test across subjects, all one-sided ps<0.05) with a gap (ΔR) of 0.43 for face-selectivity, 0.62 for body-selectivity, and 0.75 for scene-selectivity. We also visualized the multivariate response across models and subjects’ similarity with MDS (Fig. 3d). As expected, human subjects clustered closely together, showing very similar response patterns, whereas ANN models were more spread out and different from one another.

To summarize, we find that category-selective responses in the human brain are remarkably consistent across individuals by both univariate and multivariate measures. In contrast, category-selective units in ANNs differ both in their overall univariate response tuning and in the structure of their multivariate representations. Even when ANN units have the same category preference as human category-selective regions, their response patterns are measurably different. This suggests that even though category-selective units indeed exist in ANNs, their patterns of responses are highly variable across models and not brain-like.

### 2.4 Other factors do not explain differences between category-selectivity in ANNs and human brains

We identified three key experimenter-chosen parameters that could impact the comparison between category-selectivity in ANNs and human brains: 1) the selectivity threshold used to identify category-selective units and voxels, 2) the functional localizer used to identify units within ANNs, and 3) the choice of brain category-selective fROI used as the comparison target for ANN category-selective units. Next, we systematically assessed the impact of each factor on human-ANN similarity and showed that the overall pattern of results remains unchanged.

#### Category-selectivity threshold:

Applying a more stringent selectivity threshold could, in principle, yield ANN units whose responses more closely match human category-selective regions. To test this possibility, we systematically varied the selectivity thresholds used to identify category-selective ANN units and human voxels. As expected, higher thresholds produced fewer but more selective units and voxels (Fig. S5; exact paired sign-flip permutation test across subjects, all one-sided ps<0.05; lowest achieved median multivariate ΔR = 0.41, 0.56, and 0.69 for face-, body-, and scene-selectivity respectively). These results indicate that the observed ANN–brain differences cannot be explained by the selectivity threshold used to define category-selective units or voxels, and that the representational gap persists across a wide range of selectivity strengths.

#### Functional localizer:

It’s also possible that our initial localizer simply failed to identify the most brain-like category-selective units in ANNs. To rule out this possibility, we compared units identified using an alternative, widely adopted fMRI localizer [Fig. S6; one-sided Wilcoxon signed-rank test, all ps<0.0001; see Fig. S7; one-sided Wilcoxon signed-rank test on model-level differences, all ps<0.00001). The gap remained substantial across univariate (median ΔR=0.22, 0.26, and 0.22 for face-, body-, and scene-selectivity, respectively) and multivariate comparisons (median ΔR=0.5, 0.68, and 0.61 for face-, body-, and scene-selectivity, respectively). Thus, even the most brain-aligned unit subsets identifiable within current ANN models failed to reproduce the stable stimulus-level category-selective response patterns observed in human brains.

#### Choice of category-selective fROIs:

Another possibility is that category-selective ANN units may align more closely with *other* category-selective regions beyond than FFA, EBA, or PPA. To test this possibility, we compared face-, body-, and scene-selective ANN units with a broader set of 5 other category-selective fROIs (OFA, FBA, OPA, VWFA, and OVWFA, see Fig. S8; one-sided Wilcoxon signed-rank test on model-level differences, all ps<0.00001). Thus, category-selective ANN units are not brain-aligned even when comparisons extend beyond the canonical category-selective regions.

Taken together, these results show that the difference between ANN and human category selectivity is robust across analysis choices and extends beyond FFA, EBA, and PPA.

### 2.5 Category-selective ANN units are neither necessary nor sufficient for predicting brain responses

So far, our results show that category-selective ANN units do not display the stable univariate and multivariate response profiles observed in human category-selective brain regions. At the same time, we know from prior studies that ANN features can be linearly combined to predict responses in human visual cortex [56–59], including category-selective regions such as the FFA, EBA, and PPA [24, 45, 60]. One possibility is that category-selective units, even though not similar to category-selective voxels directly, are critical to the prediction accuracy of encoding models [61]. We tested this idea directly by asking: *Are category-selective units in ANNs necessary and/or sufficient for predicting category-selective responses in the brain?*

To test necessity, we performed a lesioning manipulation by removing all the previously identified category-selective ANN units (sections 2.1–2.3) and then building voxel-wise encoding models to predict responses in each target region (FFA, EBA, and PPA). We reasoned that if category-selective units were necessary for prediction, removing them should reduce model prediction scores relative to encoding models trained on all units in the layer. To test sufficiency, we trained models using *only* the category-selective units as input features. If these units were sufficient, models trained only on category-selective units should match the predictive accuracy of models trained on all units in the layer. Note that the encoding models were trained on an independent set of 485 images (shared across 4 subjects) and prediction accuracy was evaluated on the 515 held-out images used in all previous analyses (see Methods).

We found that category-selective units were neither necessary nor sufficient for the predictive accuracy of the encoding models (Fig. S9) and under a constrained sparse-positive mapping between ANN units and the brain [Fig. S10).

Together, these findings provide an independent line of evidence that category-selective ANN units do not instantiate category selectivity in the same way as the human cortex. Category-selective units in ANN models were neither necessary nor sufficient for predicting responses in human category-selective brain regions. Information necessary for brain predictivity of category-selective regions appears to be distributed broadly across ANN representations rather than concentrated within ANN units deemed category-selective.

### 2.6 Interpreting the differences between category-selectivity in ANNs and fMRI voxels

Our analyses so far show a quantitative gap between category selectivity in ANN units and brain regions. But aggregate statistical metrics do not tell us anything human-interpretable about *which* aspects of the stimulus structure are responsible for these differences. Both ANNs and brains represent information across many interacting stimulus dimensions [58, 63–69], and the differences likely arise from a combination of factors. Here, we begin to unpack this complexity by identifying some interpretable stimulus conditions patterns that elicit systematic response differences between category-selective ANN units and brain regions.

This analysis is particularly prone to circularity [Supplementary Table 1 for the list of models). This strict separation between hypothesis development and validation allowed us to assess whether the observed effects generalized to (1) held-out subjects (N=7 participants), (2) held-out stimuli (N=350 images), and (3) held-out models (N=10 models). As expected, not all our initial hypotheses held up in independent tests, and we report all outcomes below.

#### ANN face-selective units overemphasize the distinction between human and animal faces:

The clearest and most interpretable differences between ANN and brain responses emerged in the domain of face selectivity. In the exploratory phase (using the single reference model and subject), we found that face-selective ANN units showed an exaggerated difference between human and animal faces compared to the FFA (Fig. S13). Together, these results indicate that face-selective ANN units exaggerate the difference between human and animal faces compared to the human FFA, and may broadly overemphasize visual cues related to human faces.

#### ANN body-selective units are highly sensitive to limb visibility:

Based on our previous result with faces, it was natural to ask if there was a similar distinction between ANN units and the EBA for human and animal bodies. However, we found limited evidence for systematic differences under our stringent hypothesis-testing framework (Fig. S12). Instead, somewhat surprisingly, a different, more consistent pattern emerged with respect to limb visibility (Fig. S13). Together, these results suggest that ANN body-selective units place disproportionate weight on surface-level visual cues such as exposed skin, whereas the EBA exhibits a more graded and less cue-dependent representation of bodies.

#### Human scene-selective regions, and not ANN scene-selective units, are strongly suppressed by humans in scenes:

In exploratory analyses, we observed that human scene-selective responses in the PPA were strongly suppressed by the presence of people within scenes (Fig. S12). To test our hypothesis, we curated images of scenes with and without humans in them (350 total stimuli, 25 per group per subject). The PPA responded significantly less to scenes containing humans than to scenes without humans (Fig. S13). Overall, while both scene-selective ANN units and the PPA are modulated by humans within scenes, the PPA exhibits a much stronger suppression effect. In contrast, ANN scene-selective units appear less sensitive to this contextual modulation.

To summarize, these results expose consistent and interpretable differences between category selectivity in ANNs and brains. Across faces, bodies, and scenes, category-selective ANN units appear strongly influenced by the specific visual features present in the localizer stimuli. In contrast, category-selective responses in the brain generalized more consistently across stimuli and individuals. These findings suggest that category selectivity in current ANN models is comparatively fragile, relying more heavily on the particular cues used to define a category, whereas category selectivity in the brain reflects a more stable and shared representational organization.

## 3 Discussion

**Figure 4:**
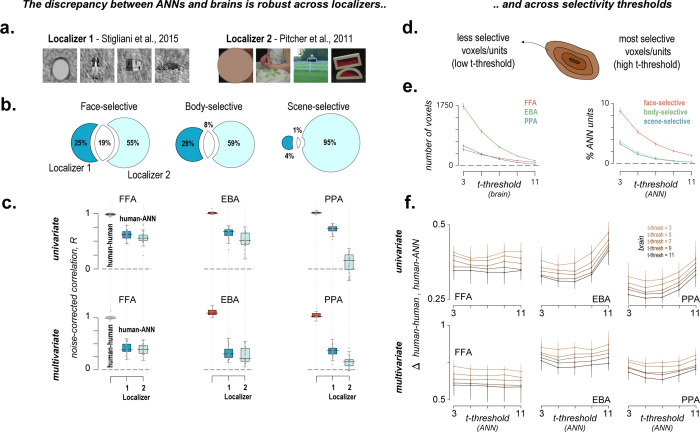
The discrepancy between selectivity in ANN units and human brains is robust across localizers and selectivity thresholds. **a.** Example images from two different functional localizers used to identify category-selective units [42, 43]. **b.** Overlap between category-selective ANN units identified by the two localizers. Percentages indicate the proportion of units unique to each localizer or shared between localizers (median across models). **c.** Human-human and human-ANN similarity for category-selective responses identified using each localizer. Human-human similarity consistently exceeded human-ANN similarity across localizers, regions, and both univariate (top) and multivariate (bottom) analyses. Each point represents one subject (human-human) or one ANN model (human-ANN) pair. **d.** Schematic illustrating the relationship between selectivity strength and the t-threshold used to define category-selective voxels and units. **e.** Effect of t-threshold on the number of category-selective voxels in the brain (left) and the proportion of category-selective units in ANNs (right). Increasing threshold (x-axis) identifies progressively more selective and fewer voxels and units (y-axis). Colors indicate each kind of selectivity. **f.** Gap between human-human and human-ANN similarity (y-axis) as a function of voxel and unit selectivity thresholds (x-axis). The discrepancy between ANN and brain category selectivity remained robust across a broad range of threshold choices for both univariate (top) and multivariate (bottom) analyses.

**Figure 5:**
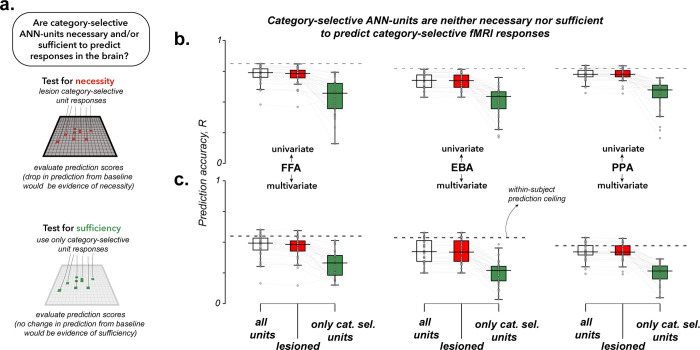
Voxel-wise encoding model predictivity from ANN units to category-selective regions is minimally impaired when ANN category-selective units are lesioned. **a** Schematic describing the question and tests to investigate whether ANN category-selective units drive the predictive accuracy of voxel-wise encoding models of category-selective regions. **b** Correlation between observed and predicted responses from voxel-wise encoding models (using ridge mapping) on held-out NSD images. For each boxplot, the x-axis indicates the unit set considered, and the y-axis indicates the correlation. The first box depicts the correlation of the encoding model using all the units within the ANN layer (each point indicating one ANN model’s median correlation across the subjects); similarly, the second and third boxes correspond to encoding models when the category-selective units are lesioned, and when only category-selective units are considered. The black dashed line shows the median within-subject ceiling, i.e., spearman-brown corrected split-half correlation between the subject’s responses during the three repetitions of the same stimuli. The two rows indicate univariate and multivariate comparisons, respectively. The three columns indicate the category-selective region, i.e., FFA, EBA, and PPA, respectively.

**Figure 6:**
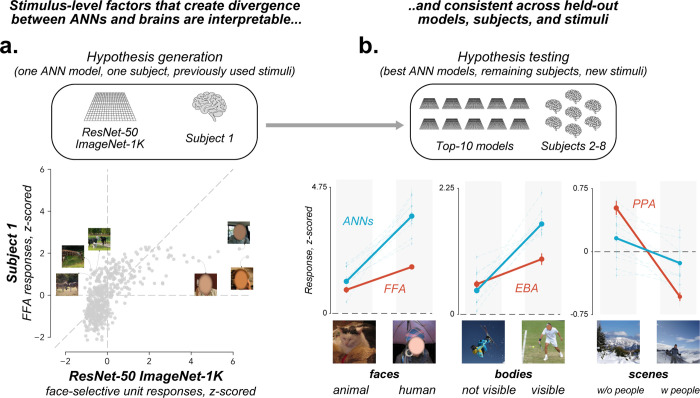
Stimulus-driven divergences between ANN and human category-selective responses generalize across held-out models, subjects, and stimuli. **a** Schematic of the hypothesis-generation framework. Hypotheses were generated using responses to the previously used evaluation subset (shared 515 NSD stimuli), from a single reference subject (S1) and a single standard model (ResNet-50 ImageNet-1K). The lower panel visualizes the identification of divergent stimuli for face selectivity. Divergent (outlier) stimuli were identified based on discrepancies between unit-averaged face-selective ANN responses (x-axis) and voxel-averaged FFA responses (y-axis), both z-scored across stimuli. NSD images are from the COCO image dataset/Flickr [74]. **b** The top panel shows a schematic of the hypothesis-validation framework. The candidate stimulus-level factors were evaluated on independent, subject-specific held-out images, held-out subjects, and held-out top-10 most brain-aligned models according to univariate tests (see Methods). The middle panel shows example NSD images from the stimulus groups that produced systematic divergence between ANN and brain responses across three domains of category selectivity: face-selective (animal faces vs. human faces), body-selective (bodies with obscured limbs vs. visible limbs), and scene-selective (scenes without humans vs. scenes with humans). The bottom panel shows a descriptive visualization of ANN–brain divergence for each stimulus group. The x-axis indicates stimulus group, and the y-axis shows unit-averaged (ANN) or voxel-averaged (brain) z-scored responses (see Methods). Three columns show divergences for face-, body-, and scene-selectivity, respectively. For each plot, the thick red lines denote the mean response across subjects (points indicate mean ± SEM), thick blue lines denote the median response across the models, and faint blue lines show individual model responses. Across all domains, ANNs exhibit robust differences between stimulus groups relative to human category-selective regions. NSD images are from the COCO image dataset/Flickr [74].

In this work, we draw the distinction between the *existence* of category selectivity in modern ANNs and the *form* of category selectivity they exhibit compared to the human brain. We first show that the stimulus-level response tuning in category-selective regions is highly consistent across individuals ($P_{2}$ for Q1, Fig. 1), and can be used as a representational benchmark for ANNs. We then applied the same localization procedure used in human fMRI to ANNs and found that face, body, and scene-selective units emerge reliably in trained, but not in untrained models (Fig. 2). Next, we compared the detailed stimulus-level response patterns and found that category-selective ANN unit responses differed substantially (both in univariate tuning and multivariate response structure, Fig. 3; $P_{1}$ for Q2, Fig. 1) relative to the human FFA, PPA, and EBA. Our results were robust to analysis choices like the functional localizer used, the selectivity threshold, and the specific regions examined (Fig. 4). Although ANN features could be linearly combined to predict voxel responses in category-selective cortex (using encoding models), lesioning experiments further confirmed that the category-selective ANN units themselves were neither necessary nor sufficient for this predictivity (Fig. 5). Finally, we used stimulus-level tests to expose some interpretable differences between category-selective ANN units and brains (Fig. 6). These findings suggest that the representations in category-selective ANN units are driven by superficial, dataset-dependent cues and differ in important ways from the consistent and structured representations observed in human category-selective brain regions.

The first key finding from our study concerns the brain itself. Category-selective regions are usually defined with coarse functional localizers that use only a small number of images and stimulus categories (typically two to five). This process raises a natural concern that the localizers might simply be grouping random voxels that pass a category contrast without otherwise a shared representational structure [75–77]. Our results suggest otherwise: category-selective regions of the brain show highly similar stimulus-level response patterns across individuals. This representational stability across individuals has several key implications. From a neuroscience perspective, it means that the fROI-based method can be used to find stable and meaningful differences between different regions (e.g., FFA vs. OFA [43, 78]), developmental stages (e.g., children vs. adults [22, 79–82]), and species (e.g., human FFA vs. ML/AL patch in macaque inferotemporal cortex [83, 84]). From a modeling perspective, this stability enables a direct comparison of selectivity in ANNs and brains. In fact, unlike the stable category selectivity observed in the human brain [46], different localizers identified very different subsets of selective units in ANNs. This suggests that selectivity in current ANN models is considerably more fragile and relies overly on the specific features present in the images used to probe the model. This observation leads to two practical recommendations for future NeuroAI research. First, studies comparing ANN units to brain regions should localize those that mirror those used in human neuroscience. Second, they should demonstrate that conclusions remain stable across different localizer choices.

The second (and central) finding of our study is that category selectivity in ANN units cannot (yet) be directly equated with the category selectivity observed with human fMRI. As we detailed in our Introduction, a category-contrast is an underconstrained test because widely different response patterns can easily satisfy it. From a model-to-brain alignment perspective, it calls for caution because the overriding category-level variance can make two systems look more similar than they really are, unless one uses more demanding analyses like comparing the noise-corrected image-grained response profiles. Therefore, finding category-selective units in ANNs should only be considered the first step. When we compared the full image-level response structure, many differences became clear. ANN units did not approach the stable level of similarity observed across human brains, either in univariate tuning or in multivariate representational structure. In many cases, ANN units were less similar to a target brain region (e.g., FFA) than other, functionally distinct category-selective regions were (e.g., EBA). Category-selective units were also highly variable across ANN models, suggesting that different models in fact converged on different solutions rather than a common brain-like form of selectivity. These mismatches extended beyond the FFA, EBA, and PPA to the broader set of category-selective regions and remained robust across localizers and selectivity thresholds. Consistent with this observation, category-selective ANN units were neither necessary nor sufficient for predicting responses in human category-selective cortex: removing these units had little effect on prediction scores, whereas restricting models to these units substantially reduced model predictivity. Taken together, these results show that ANNs can satisfy the minimal definition of category selectivity without reproducing the stable and shared form of selectivity observed in the human brain with fMRI.

Our findings open several important questions. First, what evolutionary and developmental pressures give rise to brain-like category representations? We find that ANNs trained on a wide range of tasks do not yet converge on the form of category selectivity observed in the human brain. As other studies have shown, ANNs are brain-aligned at a representational level, often more so than previous generations of models [60, 63, 85–87]. Future work will need to ask what additional constraints are required for brain-like organization to emerge. Prominent ideas suggest developmental constraints [88–91], ecological visual experience [92–95], wiring and topographic pressures [27–32], recurrent processing [96, 97], learning rules, and objectives that better capture the problems the visual system evolved to solve. Second, what determines the stability of category-selective representations? As we show, category-selective responses are highly stable across people and depend on more robust features (e.g., human vs. animal faces in FFA) [98]. As we also show, category-selective responses are much more fleeting in ANN units, often overspecialized to the features in the localizer images used to identify them. This is concerning because it shows that ANNs do not share biological invariances. Our study also gives practical guidance for how to probe the stability of these models in interpretable ways. Even simple contrasts, like human versus animal faces, scenes with versus without people, or bodies with visible versus covered limbs, are enough to reveal clear differences between voxels and ANN and can be used as diagnostic tests. Future studies that claim to find a better match between ANN units and brains should directly use these tests. Finally, our work forces us to think about the appropriate level of analysis for comparing brains and ANNs. Our results suggest that, at least in current ANN models, individual units may not be aligned to brains. Combinations of units in the meantime might be better suited for using ANN models as tools for understanding the brain [24, 45, 60, 99]. This pattern fits with prior work showing that unit-level metrics (such as RSA) often yield a weaker match to the brain than encoding models that combine features across several units [100, 101]. Future work should continue to study both levels and develop better, sharper metrics [102–105]. Population-level analyses may currently provide the strongest signal of alignment, but it will also be important to test more directly whether individual artificial units can match the response tuning of single voxels, and ultimately single neurons.

To summarize, we show that category selectivity is a much stronger test of ANN models than it initially appears. Even though current ANN models contain units that can be deemed category-selective, the form of category selectivity they exhibit remains consistently and measurably different from that observed in the human voxels.

## 4 Methods

### 4.1 fMRI data and localization details

We analyzed data from the 1.8 mm^3^ native surface preparation of the Natural Scenes Dataset (NSD; [41]), using version 3 single-trial beta estimates (betas_fithrf_GLMdenoise_RR). To reduce session-to-session variability, voxel responses were z-scored within each session and then averaged across the three repetitions of each stimulus. We focused on the 1000 images shared across subjects. Of these, 515 images were viewed three times by all 8 subjects and served as the main evaluation set. The remaining 485 images were viewed three times by a subset of 4 subjects (1, 2, 5, and 7) and were used as a training set for selecting representative ANN layers, selecting best-performing models, and fitting voxel-wise encoding models.

Our primary functional regions of interest (fROIs) included the fusiform face area (FFA; combining FFA-1 and FFA-2), extrastriate body area (EBA), and parahippocampal place area (PPA). Voxels were selected bilaterally based on category-selective responses from an independent functional localizer [42], using a contrast-specific t-value threshold. Unless otherwise noted, we used a threshold of t>7.

### 4.2 Modeling details

#### 4.2.1 Artificial Neural Network models

We analyzed 35 task-optimized artificial neural network (ANN) models spanning a diverse set of architectures, training datasets, learning objectives, and model sizes. Models were sourced from several widely used repositories, including the Torchvision (PyTorch) model zoo [Supplementary Table 1.

To assess the role of training, we additionally analyzed 20 randomly initialized (untrained) models. These consisted of four standard architectures (AlexNet, ResNet-18, ResNet-50, and ViT-B/32), each instantiated with five different random seeds (1–5). Model weights were initialized using the default PyTorch initialization procedures. The full list of untrained models is provided in Supplementary Table 2.

To extract ANN unit responses, we presented stimuli to each model and recorded activations from a given intermediate layer. Each image was first zero-padded to a square along the shorter dimension to preserve aspect ratio, then processed using the model’s native inference transforms.

#### 4.2.2 Identifying category-selective units within an ANN layer

To make the comparison with the brain as fair as possible, our goal was to use the same localization logic in ANNs that is used to identify category-selective regions in human fMRI. We therefore identified category-selective units by presenting a standard functional localizer. Our main analysis used the vpnl-fLoc localizer [42], which contains grayscale images with low-level properties matched across categories and includes 288 images each of faces, scenes, bodies, objects, and characters, along with 144 scrambled images. Note that this is the same localizer used in the Natural Scenes Dataset fMRI data used in our study [41].

We identified category-selective units by testing whether each unit responded more strongly to a preferred category than to every other category separately. For example, a face-selective unit had to respond more strongly to faces than to scenes, bodies, objects, characters, and scrambled stimuli. For each comparison, we computed a two-sample t-test, and units that exceeded the threshold in all comparisons were classified as category-selective. We repeated this procedure for face-, scene-, and body-selective units. Our main analysis used a threshold of t>7 (two-sided *p* = 7.17 × 10^−12^, df=574) for pretrained models, which was matched with the fMRI data. We report the corresponding p-values to indicate the stringency of the threshold; the threshold was used as a selection criterion to define category-selective unit populations rather than to perform statistical inference across individual units. Note that all analyses were conducted at the exemplar (single-stimulus) level, such that unit responses were evaluated on individual images rather than block-averaged category responses. This differs from standard fMRI localizer analyses, which typically rely on category-level contrasts (e.g., faces vs. non-faces in block designs); here, selectivity was defined at the level of individual stimulus responses using pairwise category comparisons, enabling a more fine-grained and conservative assessment of category preference. For untrained ANNs, several model instances had no category-selective units exceeding this stringent threshold; thus, we adopted a more permissive cutoff of t>3 (two-sided *p* = 2.82 10^−3^, df=574) to obtain reliable category-selective unit sets across all architectures and seeds.

#### 4.2.3 Layer selection

To ensure a fair comparison between ANNs and the brain, we selected, for each fROI, the layer with the highest brain–model correspondence using a data-driven criterion. To do so, we treated all intermediate feature extraction layers as candidate layers, excluding the final classification (MLP) layers. In convolutional neural networks (CNNs), this included convolutional layers, normalization and nonlinearity stages, and the final pooling layer preceding the classifier. In Transformer-based models, we considered all encoder blocks following patch and positional embeddings, and within each block, we evaluated activations at multiple stages: after the first normalization, after self-attention (before residual addition), after the second normalization, and after the MLP block (before residual addition). To preserve spatial information, individual tokens were treated independently. This procedure yielded a set of candidate layers for each model.

For a given fROI (e.g., FFA), we first identified category-selective units for the fROI’s preferred category (e.g., face-selective) in every candidate layer as described above. We then computed responses of these units to the 485-image NSD training set and compared them to voxel-averaged fROI responses from the four training subjects. Specifically, we calculated the univariate Pearson correlation between unit-averaged model responses and voxel-averaged brain responses across stimuli (see Methods 4.3). Correlations were noise-corrected to account for within-subject variability across stimulus repetitions (see Methods 4.3.1). The representative layer was defined as the layer with the maximum median noise-corrected correlation across subjects. This procedure was performed independently for each fROI, using face-, body-, and scene-selective units for FFA, EBA, and PPA, respectively.

#### 4.2.4 Selecting best-performing ANN models

To compare how well different ANN models capture category-selective responses observed in the human brain, we ranked models based on their correspondence with fMRI responses. For each model, we computed a global score using the 485-image training set. Specifically, for each fROI (FFA, EBA, and PPA), we estimated the noise-corrected univariate correlation between voxel-averaged responses and unit-averaged responses from the corresponding representative layer (see Supplementary Table 1.

#### 4.2.5 Cross-validating category-selective ANN responses

To assess whether category-selective units generalize beyond the localizer stimuli, we evaluated their responses on a held-out image set. Specifically, we measured responses of these units from the selected layer to images from a separate stimulus set [24], which contained 80 images each of faces, scenes, bodies, and objects (320 images total). Responses were averaged across units and z-scored across images. To assess the robustness of these responses, we performed a one-sided Mann–Whitney U test comparing responses to the preferred category with each non-preferred category. We also visualized the resulting unit-averaged responses for each category.

### 4.3 Comparing category-selective population responses in brains and ANNs

To compare category-selective population responses between ANNs and the human brain, we used two complementary correlation-based metrics.

#### Univariate response tuning comparison:

We compared response magnitudes across stimuli using the Pearson correlation between responses from two populations $\left(r_{1,2}\right)$. For brain data, responses were averaged across category-selective voxels, and for ANNs, across category-selective units.

To account for measurement noise within each system, we applied a noise-normalization procedure based on split-half reliability (see Methods 4.3.1). Specifically, the raw correlation between systems was divided by the square root of the product of their within-system reliabilities ($r_{1,1}$ and $r_{2,2}$), estimated from repeated presentations of the same images. This yields the noise-corrected Pearson correlation:

(1) $$\tilde{r}=\frac{r_{1,2}}{\sqrt{r_{1,1}r_{2,2}}}$$

#### Multivariate response structure comparison:

We compared the structure of population responses using representational similarity analysis (RSA) [48–50]. For each population, we constructed a response matrix across stimuli and voxels/units $\left(n_{\text{stimuli}}\times n_{\text{voxels/units}}\right)$, and computed pairwise Euclidean distances between stimuli to obtain a representational dissimilarity matrix (RDM) ($n_{\text{stimuli}}\times n_{\text{stimuli}}$; see [54] for discussion of distance metrics).

We then quantified similarity between RDMs using Spearman’s rank correlation $\left(r_{1,2}^{\text{RDM}}\right)$. As above, we applied noise normalization using split-half reliability of the RDMs ($r_{1,1}^{\text{RDM}}$ and $r_{2,2}^{\text{RDM}}$), yielding the noise-corrected RDM correlation:

(2) $${\tilde{r}}^{\text{RDM}}=\frac{r_{1,2}^{\text{RDM}}}{\sqrt{r_{1,1}^{\text{RDM}}r_{2,2}^{\text{RDM}}}}$$

Note that because both $\tilde{r}$ and ${\tilde{r}}^{\text{RDM}}$ are ratios of empirically estimated correlations, they are not strictly bounded at 1. Values slightly above 1 can occur when the between-system correlation approaches the noise ceiling and sampling variability in the reliability estimates causes the denominator to underestimate the true within-system reliability. We report values without clipping to avoid introducing downward bias.

#### 4.3.1 Estimating split-half reliabilities

To estimate the reliability of responses within each system, we computed split-half reliability for both ANN units and fMRI voxels. Because ANN models are deterministic, their unit responses are noise-free, and split-half reliability was therefore set to 1.

For voxel responses from the NSD dataset, each image was presented three times. To estimate reliability, we used a split-half procedure in which, for each repetition i∈{1,2,3} , responses from repetition i were correlated with the mean response across the other two repetitions (e.g., 1 vs. mean(2, 3), 2 vs. mean(1, 3), 3 vs. mean(1, 2)). Correlations were computed across images in the set (using voxel-averaged responses for univariate analyses and voxel-pattern correlations for multivariate analyses). Averaging across repetitions provides a more stable estimate of the underlying response pattern and reduces measurement noise.

Each raw split-half correlation was then Spearman–Brown corrected to estimate the reliability expected for the full dataset:

(3) $$r_{\text{corrected}}^{\text{split-half}}=\frac{2r_{\text{raw}}^{\text{split-half}}}{1+r_{\text{raw}}^{\text{split-half}}}.$$

This procedure yielded three corrected reliability estimates for each population. The final reliability was defined as the maximum of these estimates to mitigate downward bias from noisy repetitions, which can be substantial with few repeats.

#### 4.3.2 Establishing the human-human ceiling

To estimate the human-human ceiling, we computed noise-corrected correlations across human subjects, following the NeuroAI training test [47]. For each subject, we calculated the median correlation with all other subjects. This procedure yields a distribution of human-human similarity, which serves as an upper bound on model performance. Ceilings were estimated independently for each metric (univariate and multivariate) and each fROI.

#### 4.3.3 Comparison of human-ANN similarity to the human-human ceiling

We tested whether ANN category-selective responses reached the human-human ceiling at both the individual model level and across the population of models.

At the individual model level, for each model and subject, we computed the difference between the human-ANN noise-corrected correlation and the subject-specific inter-subject ceiling (defined as the median noise-corrected correlation between that subject and all other subjects). To test whether human-ANN similarity fell below this ceiling, we performed an exact paired sign-flip permutation test across subjects (2^8^ permutations, one-sided), using the median difference across subjects as the test statistic.

At the population level, each model was summarized by its median difference across subjects. We then tested whether these model-level differences were below zero using a one-sided Wilcoxon signed-rank test across models (N = 35).

#### 4.3.4 Comparing human-human correlations across fROIs

To contextualize the gap between model–brain similarity and the human-human ceiling for a given fROI, we compared cross-subject correlations within and across fROIs. For each subject s, we computed noise-corrected correlations between that subject’s responses in fROI_1_ and the responses of every other subject $s^{\prime }$ in both fROI_1_ and fROI_2_. For each comparison subject $s^{\prime }$, we calculated the difference between the within-region correlation (${\text{fROI}}_{1}^{s}$ vs. ${\text{fROI}}_{1}^{{\text{s}}^{\prime }}$) and the cross-region correlation (${\text{fROI}}_{1}^{s}$ vs. ${\text{fROI}}_{2}^{s^{\prime }}$). The gap for subject s was defined as the median of these differences across all comparison subjects. The final gap estimate was computed as the median of these subject-level gaps across subjects.

#### 4.3.5 Low-dimensional visualization of population response similarity

To visualize the similarity between category-selective responses in human brains and ANN models, we applied classical Multi-Dimensional Scaling (MDS). MDS embeds items in a low-dimensional space such that Euclidean distances approximate their pairwise dissimilarities. For each pair of populations (across all combinations of human subjects and ANN models), we computed noise-corrected similarity using either univariate correlations $\left(\tilde{r}\right)$ or multivariate RDM correlations $\left({\tilde{r}}^{\text{RDM}}\right)$, as described above. These similarities were converted to dissimilarities using

(4) $$d=1-\tilde{r}.$$

Dissimilarities were computed independently within each fROI (FFA, EBA, and PPA), and the final dissimilarity between each pair of populations was defined as the median across fROIs. Because noise-normalized correlations can occasionally produce negative distances due to variability in reliability estimates, we clipped all distances to a minimum value of 10^−5^ to ensure numerical stability. The resulting distance matrix (subjects and ANN models subjects and ANN models) was embedded into a two-dimensional space using classical MDS (implemented via <monospace>scikit-learn). The resulting embeddings reflect the relative dissimilarity structure of response patterns across all populations.

### 4.4 Investigating the effect of experimenter-defined parameters on category-selective response similarity between brains and ANNs

To assess how experimenter-defined choices in the analysis pipeline influence category-selective response similarity between brains and ANNs, we systematically varied key parameters, including the category-selectivity threshold and the functional localizer.

#### 4.4.1 Category-selectivity threshold

We examined how varying the category-contrast t-threshold for voxel and unit selection affected response similarity. Keeping the functional localizer fixed, we varied the threshold independently for brains and ANNs (using the previously selected representative layer) from t>3 to t>11 in increments of 2, yielding five threshold values. For each threshold, we quantified the number of selected voxels (in brains) and the proportion of selected units (in ANNs, relative to the total number of units in the layer), summarizing across subjects and models using the median. Split-half reliabilities for brain responses were re-estimated independently at each threshold. We then repeated the full analysis pipeline to compute category-selective response similarity between brains and ANNs across all pairs of thresholds (25 total pairs). To evaluate whether models reached the human-human ceiling, we applied the same statistical tests described above (see Methods 4.3.3). To visualize deviations from the ceiling, we computed a gap measure for each model, defined as the difference (median across subjects) between the model–brain correlation and the subject-specific human-human ceiling. We plotted the median gap across models for each threshold pair. As a supplementary analysis, we also report this gap for the best-performing model.

#### 4.4.2 Functional localizer

We next examined the effect of the functional localizer on category-selective unit identification. Keeping the threshold fixed t>7, we repeated the localization procedure in the previously selected representative layers of all ANN models using an alternative functional localizer [112]. This localizer consists of colored videos of faces, bodies, scenes, objects, and scrambled objects. We extracted frames from these videos to construct an image set, matching the number of images per category to the original localizer (144 scrambled; 288 per other category). To ensure diversity, we sampled five frames per video at uniform intervals (0%, 25%, 50%, 75%, and 100% of the video duration). Using this alternative localizer, we re-identified face-, scene-, and body-selective units. We then repeated the full analysis pipeline to compute category-selective response similarity between brains and ANNs using the alternative localizer.

To assess the stability of ANN category-selective unit identification across localizers, we quantified the overlap between unit sets identified using the two localizers via Intersection over Union (IoU), defined as the fraction of units shared across localizers relative to the union of identified units. We further established a ceiling for this identification procedure by measuring within-localizer consistency. Specifically, we computed a split-half IoU by dividing the stimulus set into two halves, repeating the identification procedure independently for each half, and computing the IoU between the resulting unit sets. This procedure was repeated over 50 random splits, and the final consistency estimate was taken as the median split-half IoU. Finally, to test whether across-localizer overlap was lower than within-localizer consistency, we performed a one-sided Wilcoxon signed-rank test across models for each category.

#### 4.4.3 Voxel-tuning matched ANN unit identification procedure

To test whether ANN unit subsets with more brain-like responses exist outside those identified by functional localizers, we performed a voxel–unit tuning matching analysis. For each ANN model and fROI, we considered units within the previously selected fROI-corresponding layer. Using the shared training image set (485 images), we computed the Pearson correlation between the stimulus tuning profiles of each voxel and each ANN unit across images. Correlations were noise-corrected using voxel split-half reliability (unit reliability was assumed to be 1). For each voxel, we selected the ANN unit whose responses were most strongly correlated with that voxel’s responses, yielding a one-to-one voxel–unit mapping (allowing a unit to be matched to multiple voxels). This procedure was performed independently for each subject (1, 2, 5, and 7), and the final unit subset for each model and fROI was defined as the union of units selected across subjects. We then repeated the full analysis pipeline using these matched unit subsets to estimate human-ANN response similarity and assessed whether human-ANN correlations reached the human-human ceiling using the same statistical tests described above (see Methods 4.3.3). As a control, we also evaluated a variant that enforced a unique unit assignment per voxel using a greedy matching procedure that iteratively selected the highest-correlation voxel–unit pair and removed both from further consideration. This constraint did not qualitatively affect the results.

#### 4.4.4 Choice of category-selective fROI

To assess the generality of our findings across category-selective regions, we repeated the analysis using an expanded set of fROIs in the NSD dataset, including OFA, FBA (FBA-1 and FBA-2), OPA, VWFA (VWFA-1 and VWFA-2), and OVWFA. For each fROI, voxels from both hemispheres were included. For ANNs, we used previously identified face-, body-, and scene-selective unit sets. Because word-selective units were not identified in the main analysis, we repeated the identification procedure to localize them. For each fROI, we re-identified the representative layer in all ANN models and then repeated the full analysis pipeline to estimate category-selective response similarity between brain responses and the corresponding ANN unit populations. Human-human ceilings and split-half reliabilities were estimated independently for each fROI. As before, statistical comparisons to the ceiling were performed using the same procedures described above (see Methods 4.3.3). We then tested whether model–brain correlations reached the human-human ceiling for each fROI (see Methods 4.3.3).

### 4.5 Voxel-wise encoding models

To test whether ANN unit populations can predict human brain responses, we modeled each voxel as a linear combination of ANN unit responses using an encoding model framework [45, 56–60, 85]. We considered three types of unit populations:

1. **All units**: using all the units in an ANN layer.
2. **Lesioned units**: using all units in the ANN layer except those previously localized as category-selective for the preferred category.
3. **Only category-selective units**: using only the previously localized category-selective units corresponding to the preferred category.

For each ANN model, unit populations were extracted from the previously selected layer corresponding to the target fROI. These features were used to predict voxel responses in the corresponding brain region (FFA, EBA, or PPA) for four subjects (1, 2, 5, and 7). Models were trained on the 485-image training set and evaluated on the held-out 515-image test set, as defined previously. All encoding models were fit and evaluated within each subject. For each voxel, we trained a ridge regression model (α=0.1); further optimization of α did not meaningfully change the results. Prediction accuracy was quantified using univariate Pearson correlation and multivariate RDM correlation, as defined above. Because the evaluation was performed within-subject, noise correction was not applied. Instead, split-half reliability provided a within-subject ceiling for each fROI. For each model and unit population, predictive performance was summarized as the median prediction accuracy across subjects, with 95% confidence intervals. As supplementary analyses, we repeated the pipeline across different category-selectivity thresholds (t>3 to t>11) and using sparse-positive mappings via Lasso regression with non-negative weights (α=0.01) [24, 62].

### 4.6 Interpreting the discrepancy between category-selective responses in brains and ANNs

To identify stimulus-level factors underlying discrepancies between category-selective responses in the brain and ANNs, we first constructed candidate feature groups based on systematic differences in response patterns.

For each fROI (FFA, EBA, and PPA), we examined univariate responses (voxel- or unit-averaged) from a representative subject (Subject 1) and a reference model (ResNet-50 ImageNet-1K) to the shared evaluation set (515 images). We identified groups of stimuli that elicited divergent responses between the two systems and manually extracted common visual or semantic features across these groups to generate hypotheses about the sources of these divergences.

We evaluated these hypotheses using held-out images, subjects, and models. Specifically, for each group, we selected 25 images per subject from subject-specific stimulus sets (approximately 9000 images per subject), ensuring no overlap with training or evaluation images. Only stimuli with at least one repetition were included. For each subject, responses were z-scored across the full subject-specific stimulus pool, and this normalization was applied independently to brain and ANN responses to express both relative to the same stimulus distribution. We evaluated generalization across subjects (7 held-out subjects) and models (top 25% of models based on the global score; 10 models; see Supplementary Table 1).

To quantify differences between brain and ANN responses, we fit a linear mixed-effects model for each preferred category and model. The response for trial i was modeled as

(5) $$y_{i}=\beta_{0}+\beta_{\text{cond}}{\text{Condition}}_{i}+\beta_{\text{sys}}{\text{System}}_{i}+\beta_{\text{int}}\left({\text{Condition}}_{i}\times {\text{System}}_{i}\right)+u_{s[i]}+\varepsilon_{i},$$

where ${\text{Condition}}_{i}$ denotes the stimulus group (Group 1 vs. Group 2) and ${\text{System}}_{i}$ denotes the response source (brain vs. ANN), with reference levels Group 2 and ANN. The term $u_{s[i]}\sim 𝒩\left(0,\sigma_{u}^{2}\right)$ represents a subject-specific random intercept, and $\varepsilon_{i}\sim 𝒩\left(0,\sigma^{2}\right)$ denotes residual error. The Condition × System interaction $\left(\beta_{\text{int}}\right)$ was used to assess divergence between brain and model responses. Statistical inference was based on Wald z-tests with one-sided p-values for directional hypotheses.

## Supplementary Material

Supplement 1

## Data Availability

Data and code will be made publicly available upon acceptance.

## References

[1] Kanwisher Nancy, McDermott Josh, and Chun Marvin M. “The fusiform face area: a module in human extrastriate cortex specialized for face perception”. In: Journal of neuroscience 17.11 (1997), pp. 4302–4311. 9151747 10.1523/JNEUROSCI.17-11-04302.1997 PMC6573547

[2] Puce Aina “Differential sensitivity of human visual cortex to faces, letterstrings, and textures: a functional magnetic resonance imaging study”. In: Journal of neuroscience 16.16 (1996), pp. 5205–5215. 8756449 10.1523/JNEUROSCI.16-16-05205.1996 PMC6579313

[3] McCarthy Gregory “Face-specific processing in the human fusiform gyrus”. In: Journal of cognitive neuroscience 9.5 (1997), pp. 605–610. 23965119 10.1162/jocn.1997.9.5.605

[4] Downing Paul E “A cortical area selective for visual processing of the human body”. In: Science 293.5539 (2001), pp. 2470–2473. 11577239 10.1126/science.1063414

[5] Peelen Marius V and Downing Paul E. “Selectivity for the human body in the fusiform gyrus”. In: Journal of neurophysiology 93.1 (2005), pp. 603–608. 15295012 10.1152/jn.00513.2004

[6] Epstein Russell and Kanwisher Nancy. “A cortical representation of the local visual environment”. In: Nature 392.6676 (1998), pp. 598–601. 9560155 10.1038/33402

[7] Aguirre Geoffrey K, Zarahn Eric, and D’Esposito Mark. “An area within human ventral cortex sensitive to “building” stimuli: evidence and implications”. In: Neuron 21.2 (1998), pp. 373–383. 9728918 10.1016/s0896-6273(00)80546-2

[8] Nasr Shahin “Scene-selective cortical regions in human and nonhuman primates”. In: Journal of Neuroscience 31.39 (2011), pp. 13771–13785. 21957240 10.1523/JNEUROSCI.2792-11.2011 PMC3489186

[9] Kamps Frederik S “Connectivity at the origins of domain specificity in the cortical face and place networks”. In: Proceedings of the National Academy of Sciences 117.11 (2020), pp. 6163–6169. 10.1073/pnas.1911359117 PMC7084100 32123077

[10] Cosmides Leda and Tooby John. “Origins of domain specificity: The evolution of functional organization”. In: Mapping the mind: Domain specificity in cognition and culture 853116 (1994).

[11] Ventura Paulo and Cruz Francisco. “Domain specificity vs. domain generality: The case of faces and words”. In: Vision 8.1 (2023), p. 1. 38535755 10.3390/vision8010001 PMC10974919

[12] Kanwisher Nancy. “Functional specificity in the human brain: a window into the functional architecture of the mind”. In: Proceedings of the national academy of sciences 107.25 (2010), pp. 11163–11170. 10.1073/pnas.1005062107 PMC2895137 20484679

[13] Arcaro Michael J, Schade Peter F, and Livingstone Margaret S. “Universal mechanisms and the development of the face network: what you see is what you get”. In: Annual review of vision science 5.1 (2019), pp. 341–372. 10.1146/annurev-vision-091718-014917 PMC7568401 31226011

[14] Arcaro Michael J and Livingstone Margaret S. “On the relationship between maps and domains in inferotemporal cortex”. In: Nature Reviews Neuroscience 22.9 (2021), pp. 573–583. 34345018 10.1038/s41583-021-00490-4 PMC8865285

[15] Arcaro Michael and Livingstone Margaret. “A whole-brain topographic ontology”. In: Annual Review of Neuroscience 47 (2024). 10.1146/annurev-neuro-082823-073701 PMC12337153 38360565

[16] Hesse Janis K and Tsao Doris Y. “The macaque face patch system: a turtle’s underbelly for the brain”. In: Nature Reviews Neuroscience 21.12 (2020), pp. 695–716. 33144718 10.1038/s41583-020-00393-w

[17] Tsao Doris Y and Livingstone Margaret S. “Mechanisms of face perception”. In: Annu. Rev. Neurosci. 31.1 (2008), pp. 411–437. 18558862 10.1146/annurev.neuro.30.051606.094238 PMC2629401

[18] Op de Beeck Hans P, Pillet Ineke, and Ritchie J Brendan. “Factors determining where category-selective areas emerge in visual cortex”. In: Trends in cognitive sciences 23.9 (2019), pp. 784–797. 31327671 10.1016/j.tics.2019.06.006

[19] Grill-Spector Kalanit and Weiner Kevin S. “The functional architecture of the ventral temporal cortex and its role in categorization”. In: Nature Reviews Neuroscience 15.8 (2014), pp. 536–548. 24962370 10.1038/nrn3747 PMC4143420

[20] Livingstone Margaret S, Arcaro Michael J, and Schade Peter F. “ Cortex is cortex: Ubiquitous principles drive face-domain development”. In: Trends in cognitive sciences 23.1 (2018), p. 3. 30482446 10.1016/j.tics.2018.10.009 PMC6535224

[21] Powell Lindsey J, Kosakowski Heather L, and Saxe Rebecca. “Social origins of cortical face areas”. In: Trends in cognitive sciences 22.9 (2018), pp. 752–763. 30041864 10.1016/j.tics.2018.06.009 PMC6098735

[22] Deen Ben “Organization of high-level visual cortex in human infants”. In: Nature communications 8.1 (2017), p. 13995. 10.1038/ncomms13995 PMC5234071 28072399

[23] Conway Bevil R. “The organization and operation of inferior temporal cortex”. In: Annual review of vision science 4.1 (2018), pp. 381–402. 10.1146/annurev-vision-091517-034202 PMC6404234 30059648

[24] Prince Jacob S, Alvarez George A, and Konkle Talia. “Contrastive learning explains the emergence and function of visual category-selective regions”. In: Science Advances 10.39 (2024), eadl1776. 10.1126/sciadv.adl1776 PMC11423896 39321304

[25] Baek Seungdae “Face detection in untrained deep neural networks”. In: Nature communications 12.1 (2021), p. 7328. 10.1038/s41467-021-27606-9 PMC8677765 34916514

[26] Lu Zitong and Wang Yuxin. “Category-Selective Neurons in Deep Networks: Comparing Purely Visual and Visual-Language Models”. In: arXiv preprint arXiv:2502.16456 (2025).

[27] T Anderson Keller Qinghe Gao, and Welling Max. “Modeling category-selective cortical regions with topographic variational autoencoders”. In: arXiv preprint arXiv:2110.13911 (2021).

[28] Lee Hyodong “Topographic deep artificial neural networks reproduce the hallmarks of the primate inferior temporal cortex face processing network”. In: BioRxiv (2020), pp. 2020–07.

[29] Deb Mayukh, Deb Mainak, and Murty N. “TopoNets: High performing vision and language models with brain-like topography”. In: arXiv preprint arXiv:2501.16396 (2025). PMC12964247 41799785

[30] Margalit Eshed “A unifying framework for functional organization in early and higher ventral visual cortex”. In: Neuron 112.14 (2024), pp. 2435–2451. 38733985 10.1016/j.neuron.2024.04.018 PMC11257790

[31] Qian Xinyu “Local lateral connectivity is sufficient for replicating cortex-like topographical organization in deep neural networks”. In: bioRxiv (2024), pp. 2024–08. 10.1038/s41467-026-70065-3 PMC13139385 41839855

[32] Blauch Nicholas M, Behrmann Marlene, and Plaut David C. “A connectivity-constrained computational account of topographic organization in primate high-level visual cortex”. In: Proceedings of the National Academy of Sciences 119.3 (2022), e2112566119. 10.1073/pnas.2112566119 PMC8784138 35027449

[33] Doshi Fenil R and Konkle Talia. “Cortical topographic motifs emerge in a self-organized map of object space”. In: Science Advances 9.25 (2023), eade8187. 10.1126/sciadv.ade8187 PMC10284546 37343093

[34] Lu Zejin “End-to-end topographic networks as models of cortical map formation and human visual behaviour”. In: Nature Human Behaviour (2025), pp. 1–17. 10.1038/s41562-025-02220-7 PMC12454150 40481218

[35] Nicholas M Blauch Marlene Behrmann, and Plaut David C. Retinotopic scaffolding of high-level vision. 2025. doi: 10.31234/osf.io/rynbz_v2. url: osf.io/preprints/psyarxiv/rynbz_v2.

[36] Zhou Deming “TDSNNs: Competitive Topographic Deep Spiking Neural Networks for Visual Cortex Modeling”. In: arXiv preprint arXiv:2508.04270 (2025).

[37] Wang Jinge “Face identity coding in the deep neural network and primate brain”. In: Communications Biology 5.1 (2022), p. 611. 35725902 10.1038/s42003-022-03557-9 PMC9209415

[38] Honarmand Melika “Inducing Dyslexia in Vision Language Models”. In: arXiv preprint arXiv:2509.24597 (2025).

[39] Fisher Matthew and Keil Frank C. “The binary bias: A systematic distortion in the integration of information”. In: Psychological Science 29.11 (2018), pp. 1846–1858. 30285536 10.1177/0956797618792256

[40] MacCallum Robert C “ On the practice of dichotomization of quantitative variables.” In: Psychological methods 7.1 (2002), p. 19. 11928888 10.1037/1082-989x.7.1.19

[41] Allen Emily J “A massive 7T fMRI dataset to bridge cognitive neuroscience and artificial intelligence”. In: Nature neuroscience 25.1 (2022), pp. 116–126. 34916659 10.1038/s41593-021-00962-x

[42] Stigliani Anthony, Weiner Kevin S, and Grill-Spector Kalanit. “Temporal processing capacity in high-level visual cortex is domain specific”. In: Journal of Neuroscience 35.36 (2015), pp. 12412–12424. 26354910 10.1523/JNEUROSCI.4822-14.2015 PMC4563034

[43] Pitcher David “Differential selectivity for dynamic versus static information in face-selective cortical regions”. In: Neuroimage 56.4 (2011), pp. 2356–2363. 21473921 10.1016/j.neuroimage.2011.03.067

[44] Cohen Michael A “Visual search for object categories is predicted by the representational architecture of high-level visual cortex”. In: Journal of neurophysiology 117.1 (2017), pp. 388–402. 27832600 10.1152/jn.00569.2016 PMC5236111

[45] Murty N Apurva Ratan “Computational models of category-selective brain regions enable high-throughput tests of selectivity”. In: Nature communications 12.1 (2021), p. 5540. 10.1038/s41467-021-25409-6 PMC8452636 34545079

[46] Molloy M Fiona, Saygin Zeynep M, and Osher David E. “Predicting high-level visual areas in the absence of task fMRI”. In: Scientific reports 14.1 (2024), p. 11376. 38762549 10.1038/s41598-024-62098-9 PMC11102456

[47] Feather Jenelle “Brain-Model Evaluations Need the NeuroAI Turing Test”. In: arXiv preprint arXiv:2502.16238 (2025).

[48] Kriegeskorte Nikolaus, Mur Marieke, and Bandettini Peter A. “Representational similarity analysis-connecting the branches of systems neuroscience”. In: Frontiers in systems neuroscience 2 (2008), p. 249. 10.3389/neuro.06.004.2008 PMC2605405 19104670

[49] Mur Marieke, Bandettini Peter A, and Kriegeskorte Nikolaus. “Revealing representational content with pattern-information fMRI—an introductory guide”. In: Social cognitive and affective neuroscience 4.1 (2009), pp. 101–109. 19151374 10.1093/scan/nsn044 PMC2656880

[50] Diedrichsen Jörn and Kriegeskorte Nikolaus. “Representational models: A common framework for understanding encoding, pattern-component, and representational-similarity analysis”. In: PLoS computational biology 13.4 (2017), e1005508. 10.1371/journal.pcbi.1005508 PMC5421820 28437426

[51] Haxby James V “ Distributed and overlapping representations of faces and objects in ventral temporal cortex”. In: Science 293.5539 (2001), pp. 2425–2430. 10.1126/science.1063736 11577229

[52] Haxby James V, Connolly Andrew C, and Guntupalli J Swaroop. “Decoding neural representational spaces using multivariate pattern analysis”. In: Annual review of neuroscience 37.1 (2014), pp. 435–456. 10.1146/annurev-neuro-062012-170325 25002277

[53] Norman Kenneth A “Beyond mind-reading: multi-voxel pattern analysis of fMRI data”. In: Trends in cognitive sciences 10.9 (2006), pp. 424–430. 16899397 10.1016/j.tics.2006.07.005

[54] Walther Alexander “Reliability of dissimilarity measures for multi-voxel pattern analysis”. In: Neuroimage 137 (2016), pp. 188–200. 26707889 10.1016/j.neuroimage.2015.12.012

[55] Arend Luke Single units in a deep neural network functionally correspond with neurons in the brain: preliminary results. Tech. rep. Center for Brains, Minds and Machines (CBMM), 2018.

[56] Kay Kendrick N “Identifying natural images from human brain activity”. In: Nature 452.7185 (2008), pp. 352–355. 18322462 10.1038/nature06713 PMC3556484

[57] Naselaris Thomas “Encoding and decoding in fMRI”. In: Neuroimage 56.2 (2011), pp. 400–410. 20691790 10.1016/j.neuroimage.2010.07.073 PMC3037423

[58] Huth Alexander G “A continuous semantic space describes the representation of thousands of object and action categories across the human brain”. In: Neuron 76.6 (2012), pp. 1210–1224. 23259955 10.1016/j.neuron.2012.10.014 PMC3556488

[59] Güçlü Umut and Van Gerven Marcel AJ. “Deep neural networks reveal a gradient in the complexity of neural representations across the ventral stream”. In: Journal of Neuroscience 35.27 (2015), pp. 10005–10014. 26157000 10.1523/JNEUROSCI.5023-14.2015 PMC6605414

[60] Conwell Colin “A large-scale examination of inductive biases shaping high-level visual representation in brains and machines”. In: Nature Communications 15.1 (2024), p. 9383. 10.1038/s41467-024-53147-y PMC11526138 39477923

[61] AlKhamissi Badr “The llm language network: A neuroscientific approach for identifying causally task-relevant units”. In: Proceedings of the 2025 Conference of the Nations of the Americas Chapter of the Association for Computational Linguistics: Human Language Technologies (Volume 1: Long Papers). 2025, pp. 10887–10911.

[62] Prince Jacob S “A case for sparse positive alignment of neural systems”. In: ICLR 2024 Workshop on Representational Alignment. 2024.

[63] Chen Zirui and Bonner Michael F. “Universal dimensions of visual representation”. In: Science Advances 11.27 (2025), eadw7697. 10.1126/sciadv.adw7697 PMC12219468 40601729

[64] Groen Iris IA “Distinct contributions of functional and deep neural network features to representational similarity of scenes in human brain and behavior”. In: Elife 7 (2018), e32962. 10.7554/eLife.32962 PMC5860866 29513219

[65] Hebart Martin N “Revealing the multidimensional mental representations of natural objects underlying human similarity judgements”. In: Nature human behaviour 4.11 (2020), pp. 1173–1185. 10.1038/s41562-020-00951-3 PMC7666026 33046861

[66] Meenakshi Khosla N Murty Apurva Ratan, and Kanwisher Nancy. “A highly selective response to food in human visual cortex revealed by hypothesis-free voxel decomposition”. In: Current Biology 32.19 (2022), pp. 4159–4171. 36027910 10.1016/j.cub.2022.08.009 PMC9561032

[67] Elmoznino Eric and Bonner Michael F. “High-performing neural network models of visual cortex benefit from high latent dimensionality”. In: PLoS computational biology 20.1 (2024), e1011792. 10.1371/journal.pcbi.1011792 PMC10805290 38198504

[68] Gauthaman Raj Magesh and Bonner Michael F. “ Shared high-dimensional latent structure in the neural and mental representations of objects”. In: ().

[69] Han Chihye and Bonner Michael F. “High-dimensional structure underlying individual differences in naturalistic visual experience”. In: Current Biology 36.3 (2026), pp. 723–733. 41570814 10.1016/j.cub.2025.12.039

[70] Kriegeskorte Nikolaus “Circular analysis in systems neuroscience: the dangers of double dipping”. In: Nature neuroscience 12.5 (2009), pp. 535–540. 19396166 10.1038/nn.2303 PMC2841687

[71] Kanwisher Nancy, Stanley Damian, and Harris Alison. “The fusiform face area is selective for faces not animals”. In: Neuroreport 10.1 (1999), pp. 183–187. 10094159 10.1097/00001756-199901180-00035

[72] Grill-Spector Kalanit, Sayres Rory, and Ress David. “High-resolution imaging reveals highly selective nonface clusters in the fusiform face area”. In: Nature neuroscience 9.9 (2006), pp. 1177–1185. 16892057 10.1038/nn1745

[73] Kaiser Daniel, Azzalini Damiano C, and Peelen Marius V. “Shape-independent object category responses revealed by MEG and fMRI decoding”. In: Journal of neurophysiology 115.4 (2016), pp. 2246–2250. 26740535 10.1152/jn.01074.2015 PMC4869498

[74] Lin Tsung-Yi “Microsoft coco: Common objects in context”. In: Computer vision–ECCV 2014: 13th European conference, zurich, Switzerland, September 6–12, 2014, proceedings, part v 13. Springer. 2014, pp. 740–755.

[75] Ritchie J Brendan “Rethinking category-selectivity in human visual cortex”. In: Cognitive neuroscience (2025), pp. 1–28. 40836402 10.1080/17588928.2025.2543890 PMC12458057

[76] Vogels Rufin. “Rethinking category selectivity: insights from the macaque inferior temporal cortex”. In: Cognitive Neuroscience 17.2 (2026), pp. 106–108. 41257481 10.1080/17588928.2025.2590658

[77] Bardon Alexandra “Face neurons encode nonsemantic features”. In: Proceedings of the national academy of sciences 119.16 (2022), e2118705119. 10.1073/pnas.2118705119 PMC9169805 35377737

[78] Grill-Spector Kalanit “The functional neuroanatomy of human face perception”. In: Annual review of vision science 3 (2017), pp. 167–196. 10.1146/annurev-vision-102016-061214 PMC6345578 28715955

[79] O’Doherty Cliona “Infants have rich visual categories in ventrotemporal cortex at 2 months of age”. In: Nature Neuroscience (2026), pp. 1–10. 10.1038/s41593-025-02187-8 PMC12971487 41629539

[80] Scherf K Suzanne “Visual category-selectivity for faces, places and objects emerges along different developmental trajectories”. In: Developmental science 10.4 (2007), F15–F30. 17552930 10.1111/j.1467-7687.2007.00595.x

[81] Nordt Marisa “Cortical recycling in high-level visual cortex during childhood development”. In: Nature human behaviour 5.12 (2021), pp. 1686–1697. 10.1038/s41562-021-01141-5 PMC8678383 34140657

[82] Kosakowski Heather L “Selective responses to faces, scenes, and bodies in the ventral visual pathway of infants”. In: Current Biology 32.2 (2022), pp. 265–274. 34784506 10.1016/j.cub.2021.10.064 PMC8792213

[83] Vinken Kasper, Sharma Saloni, and Livingstone Margaret S. “ Mapping macaque to human cortex with natural scene responses”. In: Proceedings of the National Academy of Sciences 122.40 (2025), e2512619122. 10.1073/pnas.2512619122 PMC12519150 41026824

[84] Tsao Doris Y, Moeller Sebastian, and Freiwald Winrich A. “Comparing face patch systems in macaques and humans”. In: Proceedings of the National Academy of Sciences 105.49 (2008), pp. 19514–19519. 10.1073/pnas.0809662105 PMC2614792 19033466

[85] Yamins Daniel LK “ Performance-optimized hierarchical models predict neural responses in higher visual cortex”. In: Proceedings of the national academy of sciences 111.23 (2014), pp. 8619–8624. 10.1073/pnas.1403112111 PMC4060707 24812127

[86] Khaligh-Razavi Seyed-Mahdi and Kriegeskorte Nikolaus. “Deep supervised, but not unsupervised, models may explain IT cortical representation”. In: PLoS computational biology 10.11 (2014), e1003915. 10.1371/journal.pcbi.1003915 PMC4222664 25375136

[87] Schrimpf Martin “Integrative benchmarking to advance neurally mechanistic models of human intelligence”. In: Neuron 108.3 (2020), pp. 413–423. 32918861 10.1016/j.neuron.2020.07.040

[88] Jang Hojin and Tong Frank. “Improved modeling of human vision by incorporating robustness to blur in convolutional neural networks”. In: Nature Communications 15.1 (2024), p. 1989. 10.1038/s41467-024-45679-0 PMC10915141 38443349

[89] Vogelsang Marin “Impact of early visual experience on later usage of color cues”. In: Science 384.6698 (2024), pp. 907–912. 38781366 10.1126/science.adk9587 PMC12226138

[90] Lu Zejin “Adopting a human developmental visual diet yields robust and shape-based AI vision”. In: Nature Machine Intelligence (2026), pp. 1–14.

[91] Cai Yusen “Learning to See Through a Baby’s Eyes: Early Visual Diets Enable Robust Visual Intelligence in Humans and Machines”. In: arXiv preprint arXiv:2511.14440 (2025).

[92] Mehrer Johannes “An ecologically motivated image dataset for deep learning yields better models of human vision”. In: Proceedings of the National Academy of Sciences 118.8 (2021), e2011417118. 10.1073/pnas.2011417118 PMC7923360 33593900

[93] Bambach Sven “Toddler-inspired visual object learning”. In: Advances in neural information processing systems 31 (2018).

[94] Long Bria “The BabyView dataset: High-resolution egocentric videos of infants’ and young children’s everyday experiences”. In: arXiv preprint arXiv:2406.10447 (2024).

[95] Orhan A Emin and Lake Brenden M. “Learning high-level visual representations from a child’s perspective without strong inductive biases”. In: Nature Machine Intelligence 6.3 (2024), pp. 271–283.

[96] Kubilius Jonas “Brain-like object recognition with high-performing shallow recurrent ANNs”. In: Advances in neural information processing systems 32 (2019).

[97] Kietzmann Tim C “Recurrence is required to capture the representational dynamics of the human visual system”. In: Proceedings of the National Academy of Sciences 116.43 (2019), pp. 21854–21863. 10.1073/pnas.1905544116 PMC6815174 31591217

[98] van Dyck Leonard E, Hebart Martin N, and Dobs Katharina. “Multidimensional feature tuning in category-selective areas of human visual cortex”. In: bioRxiv (2025), pp. 2025–06. 10.1523/JNEUROSCI.0038-26.2026 PMC13446086 42309813

[99] Gifford Alessandro T “The algonauts project 2025 challenge: How the human brain makes sense of multimodal movies”. In: arXiv preprint arXiv:2501.00504 (2024).

[100] Soni Ansh “Conclusions about neural network to brain alignment are profoundly impacted by the similarity measure”. In: bioRxiv (2024), pp. 2024–08.

[101] Xu Yaoda and Vaziri-Pashkam Maryam. “Limits to visual representational correspondence between convolutional neural networks and the human brain”. In: Nature communications 12.1 (2021), p. 2065. 10.1038/s41467-021-22244-7 PMC8024324 33824315

[102] Kriegeskorte Nikolaus and Diedrichsen Jörn. “Peeling the onion of brain representations”. In: Annual review of neuroscience 42.1 (2019), pp. 407–432. 10.1146/annurev-neuro-080317-061906 31283895

[103] Khosla Meenakshi and Williams Alex H. “Soft matching distance: A metric on neural representations that captures single-neuron tuning”. In: Proceedings of unireps: the first workshop on unifying representations in neural models. PMLR. 2024, pp. 326–341.

[104] Khosla Meenakshi “Privileged representational axes in biological and artificial neural networks”. In: bioRxiv (2024), pp. 2024–06.

[105] Kapoor Chaitanya, Williams Alex H, and Khosla Meenakshi. “Partial Soft-Matching Distance for Neural Representational Comparison with Partial Unit Correspondence”. In: arXiv preprint arXiv:2602.19331 (2026).

[106] Paszke Adam “Pytorch: An imperative style, high-performance deep learning library”. In: Advances in neural information processing systems 32 (2019).

[107] Wightman Ross. PyTorch Image Models. https://github.com/rwightman/pytorch-image-models. 2019. doi: 10.5281/zenodo.4414861.

[108] Ilharco Gabriel OpenCLIP. Version 0.1. If you use this software, please cite it as below. July 2021. doi: 10.5281/zenodo.5143773. url: https://doi.org/10.5281/zenodo.5143773.

[109] Radford Alec “Learning transferable visual models from natural language supervision”. In: International conference on machine learning. PmLR. 2021, pp. 8748–8763.

[110] Konkle Talia and Alvarez George A. “A self-supervised domain-general learning framework for human ventral stream representation”. In: Nature communications 13.1 (2022), p. 491. 10.1038/s41467-022-28091-4 PMC8789817 35078981

[111] Wolf Thomas “Transformers: State-of-the-Art Natural Language Processing”. In: Proceedings of the 2020 Conference on Empirical Methods in Natural Language Processing: System Demonstrations. Online: Association for Computational Linguistics, Oct. 2020, pp. 38–45. url: https://www.aclweb.org/anthology/2020.emnlpdemos.6.

[112] Julian Joshua B “An algorithmic method for functionally defining regions of interest in the ventral visual pathway”. In: Neuroimage 60.4 (2012), pp. 2357–2364. 10.1016/j.neuroimage.2012.02.055 22398396

[113] Tschannen Michael “Siglip 2: Multilingual vision-language encoders with improved semantic understanding, localization, and dense features”. In: arXiv preprint arXiv:2502.14786 (2025).

[114] Liu Zhuang “A convnet for the 2020s”. In: Proceedings of the IEEE/CVF conference on computer vision and pattern recognition. 2022, pp. 11976–11986.

[115] Darcet Timothée “Vision transformers need registers”. In: arXiv preprint arXiv:2309.16588 (2023).

[116] Chen Ting “A simple framework for contrastive learning of visual representations”. In: International conference on machine learning. PmLR. 2020, pp. 1597–1607.

[117] He Kaiming “Deep residual learning for image recognition”. In: Proceedings of the IEEE conference on computer vision and pattern recognition. 2016, pp. 770–778.

[118] Oquab Maxime “Dinov2: Learning robust visual features without supervision”. In: arXiv preprint arXiv:2304.07193 (2023).

[119] Yerxa Thomas “Contrastive-equivariant self-supervised learning improves alignment with primate visual area it”. In: Advances in neural information processing systems 37 (2024), pp. 96045–96070. 40336515 PMC12058038

[120] Yang Kaiyu “A study of face obfuscation in imagenet”. In: International Conference on Machine Learning. PMLR. 2022, pp. 25313–25330.

[121] Fang Yuxin “Eva-02: A visual representation for neon genesis”. In: Image and Vision Computing (2024), p. 105171.

[122] Peng Zhiliang “Kosmos-2: Grounding multimodal large language models to the world”. In: arXiv preprint arXiv:2306.14824 (2023).

[123] Tian Yonglong “Learning vision from models rivals learning vision from data”. In: Proceedings of the IEEE/CVF conference on computer vision and pattern recognition. 2024, pp. 15887–15898.

[124] Simonyan Karen and Zisserman Andrew. “ Very deep convolutional networks for large-scale image recognition”. In: arXiv preprint arXiv:1409.1556 (2014).

[125] Guo Chong “Adversarially trained neural representations may already be as robust as corresponding biological neural representations”. In: arXiv preprint arXiv:2206.11228 (2022).

[126] Fu Stephanie “Dreamsim: Learning new dimensions of human visual similarity using synthetic data”. In: arXiv preprint arXiv:2306.09344 (2023).

[127] Geirhos Robert “ImageNet-trained CNNs are biased towards texture; increasing shape bias improves accuracy and robustness”. In: International conference on learning representations. 2018.

[128] Brendel Wieland and Bethge Matthias. “Approximating cnns with bag-of-local-features models works surprisingly well on imagenet”. In: arXiv preprint arXiv:1904.00760 (2019).

[129] Dosovitskiy Alexey. “An image is worth 16×16 words: Transformers for image recognition at scale”. In: arXivpreprint arXiv:2010.11929 (2020).

[130] Krizhevsky Alex, Sutskever Ilya, and Hinton Geoffrey E. “Imagenet classification with deep convolutional neural networks”. In: Advances in neural information processing systems 25 (2012).

[131] Zbontar Jure “Barlow twins: Self-supervised learning via redundancy reduction”. In: International conference on machine learning. PMLR. 2021, pp. 12310–12320.

[132] Ridnik Tal “Imagenet-21k pretraining for the masses”. In: arXiv preprint arXiv:2104.10972 (2021).

