# Supplementary material for: Category selectivity observed in the human brain is distinct from category selectivity observed in artificial neural networks
Source: bioRxiv. 2026 Jun 2:2026.05.29.728609. Preprint. [Version 1] doi: 10.64898/2026.05.29.728609 (PMC13252086; doi:10.64898/2026.05.29.728609)
Supplement: Supplement 1 [file NIHPP2026.05.29.728609v1-supplement-1.pdf]

bioRxiv preprint doi: https://doi.org/10.64898/2026.05.29.728609; this version posted June 2, 2026. The copyright holder for this preprint (which was not certified by peer review) is the author/funder, who has granted bioRxiv a license to display the preprint in perpetuity. It is made available under a CC-BY 4.0 International license.

5 Supplemental Information

Supplemental Figures

S1. Trained ANN models have robust face-, body- and scene-selective units

S2. Untrained ANN models do not have robust face-, body- and scene-selective units

S3. Noise-corrected univariate correlations across all subjects and models, for all category-selective regions

S4. Noise-corrected multivariate correlations across all subjects and models, for all category-selective regions

S5. Univariate and multivariate correlation gaps between brains and best-performing model across combinations of brain and ANN selectivity thresholds

S6. Intersection of unit sets identified within and across different localizers

S7. Noise-corrected univariate and multivariate correlations for voxel-matched ANN unit subsets

S8. Noise-corrected univariate and multivariate correlations across an extended set of category-selective regions.

S9. Prediction accuracy of voxel-wise encoding models when category-selective units are lesioned, across selectivity thresholds

S10. Prediction accuracy of voxel-wise encoding models when category-selective units are lesioned, using sparse-positive mapping

S11. Brain and ANN responses for chosen stimulus groups, across all selectivities

S12. Brain and ANN responses for extended stimulus groups, across all selectivities

S13. Brain and ANN responses for all stimulus groups, including random, across all selectivities

Supplemental Tables

S1. Summary of all the pretrained models evaluated

S2. Summary of all the untrained model architectures evaluated

bioRxiv preprint doi: https://doi.org/10.64898/2026.05.29.728609; this version posted June 2, 2026. The copyright holder for this preprint (which was not certified by peer review) is the author/funder, who has granted bioRxiv a license to display the preprint in perpetuity. It is made available under a CC-BY 4.0 International license.

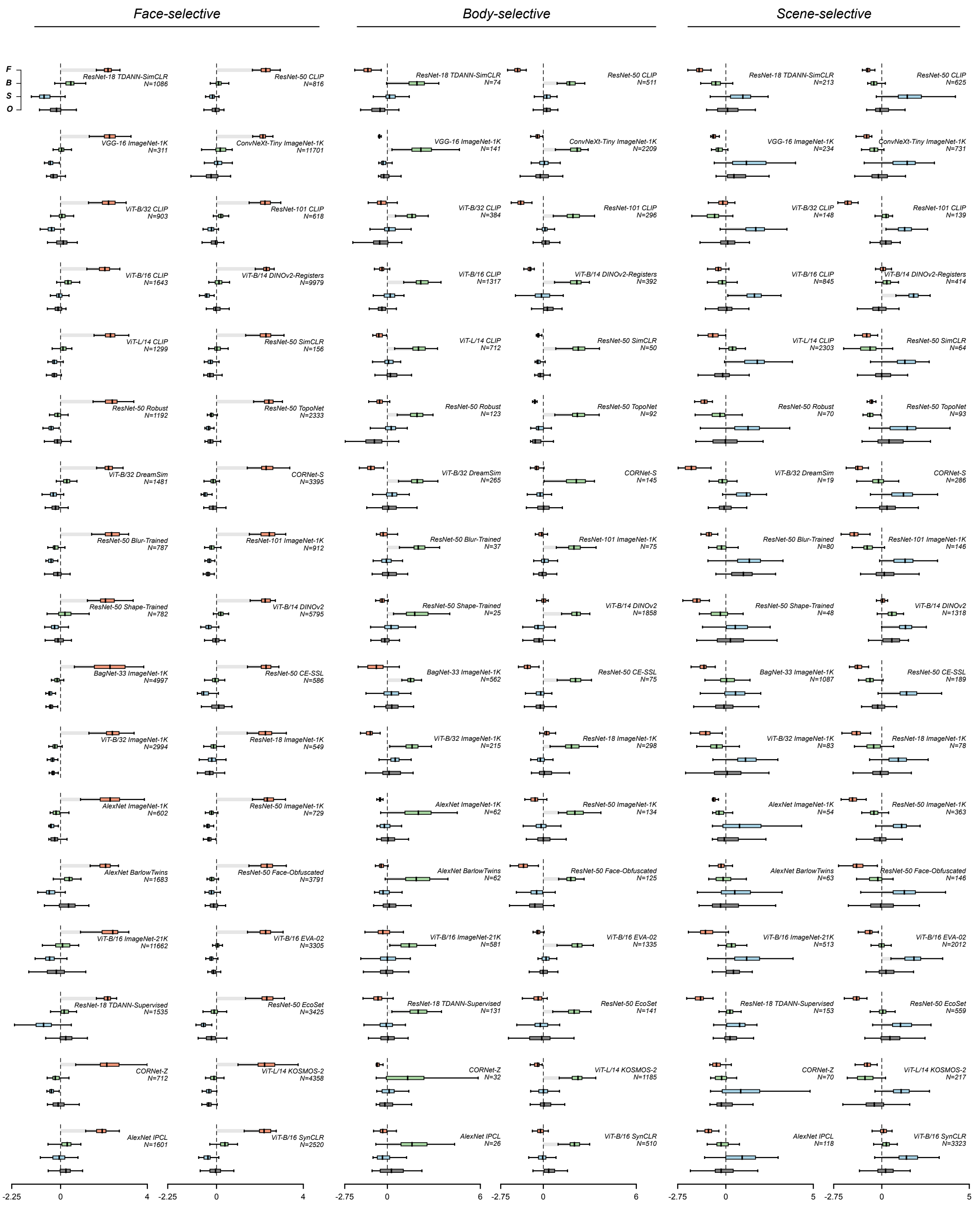

Figure S1 Trained ANN models have robust face-, body- and scene-selective units. Responses of localized face-, body-, and scene-selective units to independent stimuli of faces (F), bodies (B), scenes (S), and objects (O). For all boxplots, the y-axis indicates the stimulus category, and the x-axis shows the unit-averaged z-scored responses for a model. The annotated text shows the model name and the number of units localized. The grouped columns show responses of face-, body-, and scene-selective units, respectively. The best-performing model (ViT-B/16 SigLIP2) is repeated from Fig. 2.

bioRxiv preprint doi: https://doi.org/10.64898/2026.05.29.728609; this version posted June 2, 2026. The copyright holder for this preprint (which was not certified by peer review) is the author/funder, who has granted bioRxiv a license to display the preprint in perpetuity. It is made available under a CC-BY 4.0 International license.

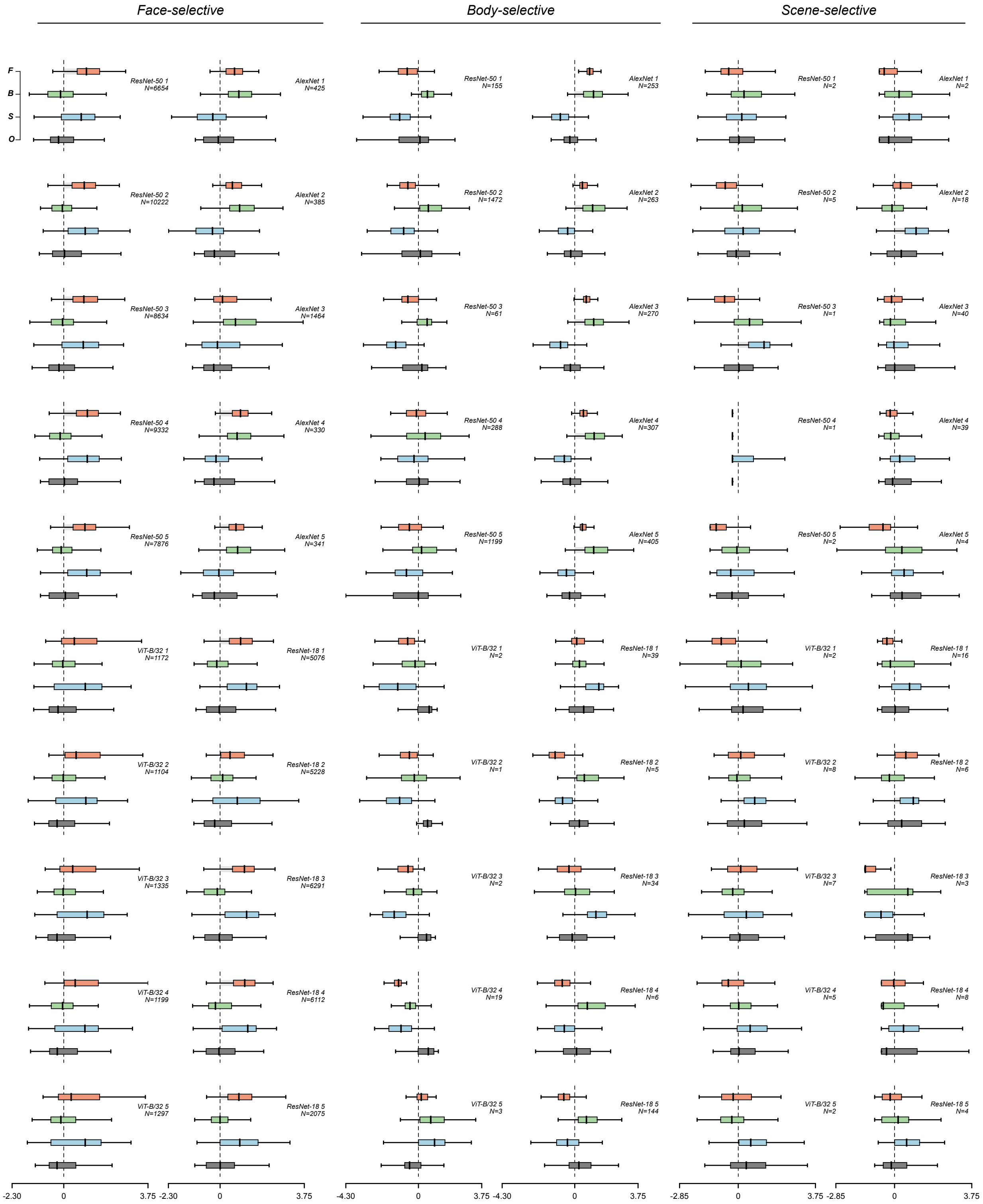

Figure S2 Untrained ANN models do not have robust face-, body- and scene-selective units. Responses of localized face-, body-, and scene-selective units to independent stimuli of faces (F), bodies (B), scenes (S), and objects (O). For all boxplots, the y-axis indicates the stimulus category, and the x-axis shows the unit-averaged z-scored responses for a model. The annotated text shows the model name and the number of units localized. The grouped columns show responses of face-, body-, and scene-selective units, respectively. AlexNet 5 is repeated from Fig. 2.

bioRxiv preprint doi: https://doi.org/10.64898/2026.05.29.728609; this version posted June 2, 2026. The copyright holder for this preprint (which was not certified by peer review) is the author/funder, who has granted bioRxiv a license to display the preprint in perpetuity. It is made available under a CC-BY 4.0 International license.

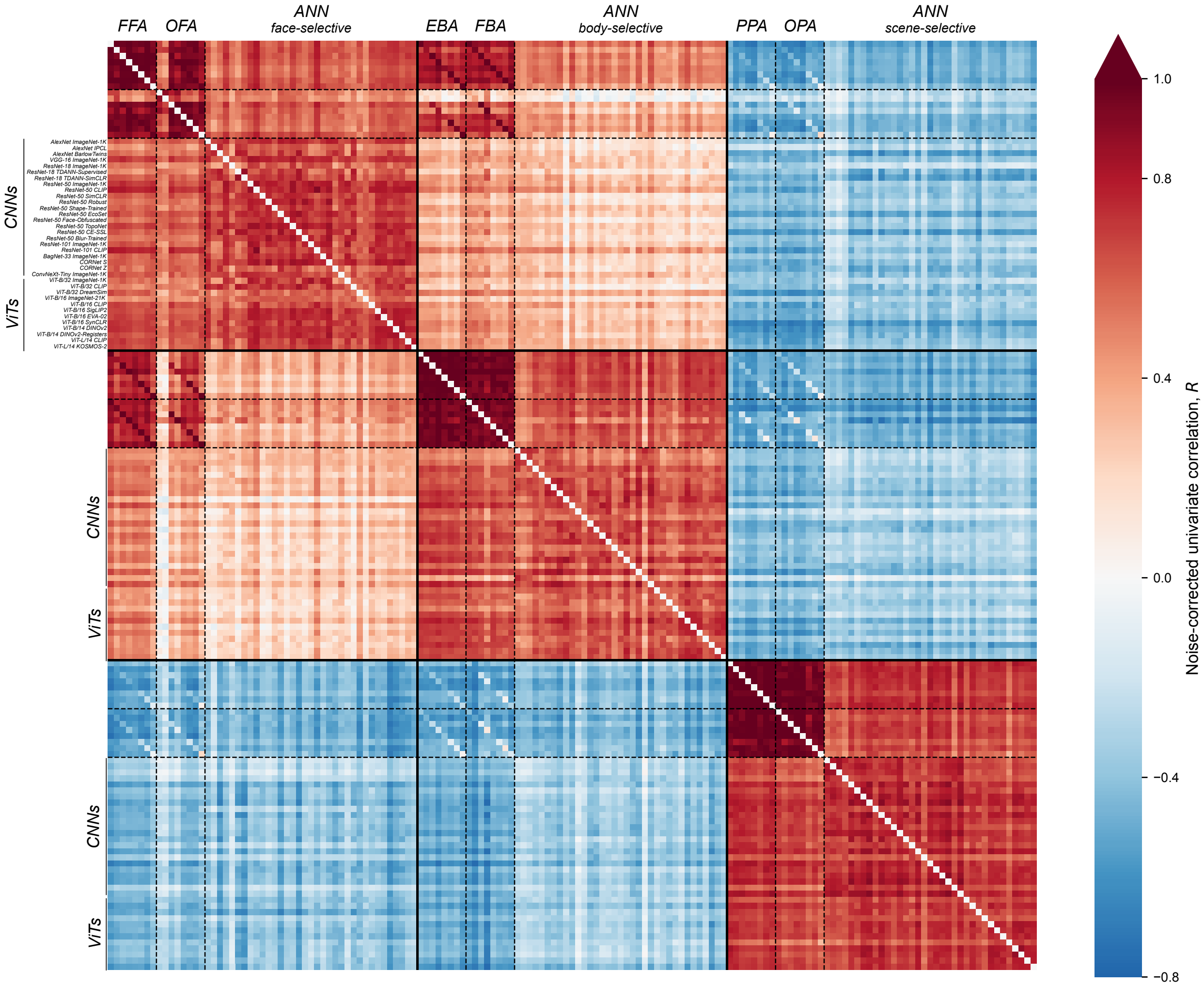

Figure S3 Noise-corrected univariate correlations across all subjects and trained models, for all category-selective regions. Noise-corrected univariate correlations between all pairs of subjects and models, across all selectivities and an extended set of category-selective regions. ANN representative layers correspond to canonical category-selective regions, i.e., FFA, EBA, and PPA. Subjects are arranged numerically (1-8), and models are grouped based on their architectures, with model names annotated.

bioRxiv preprint doi: https://doi.org/10.64898/2026.05.29.728609; this version posted June 2, 2026. The copyright holder for this preprint (which was not certified by peer review) is the author/funder, who has granted bioRxiv a license to display the preprint in perpetuity. It is made available under a CC-BY 4.0 International license.

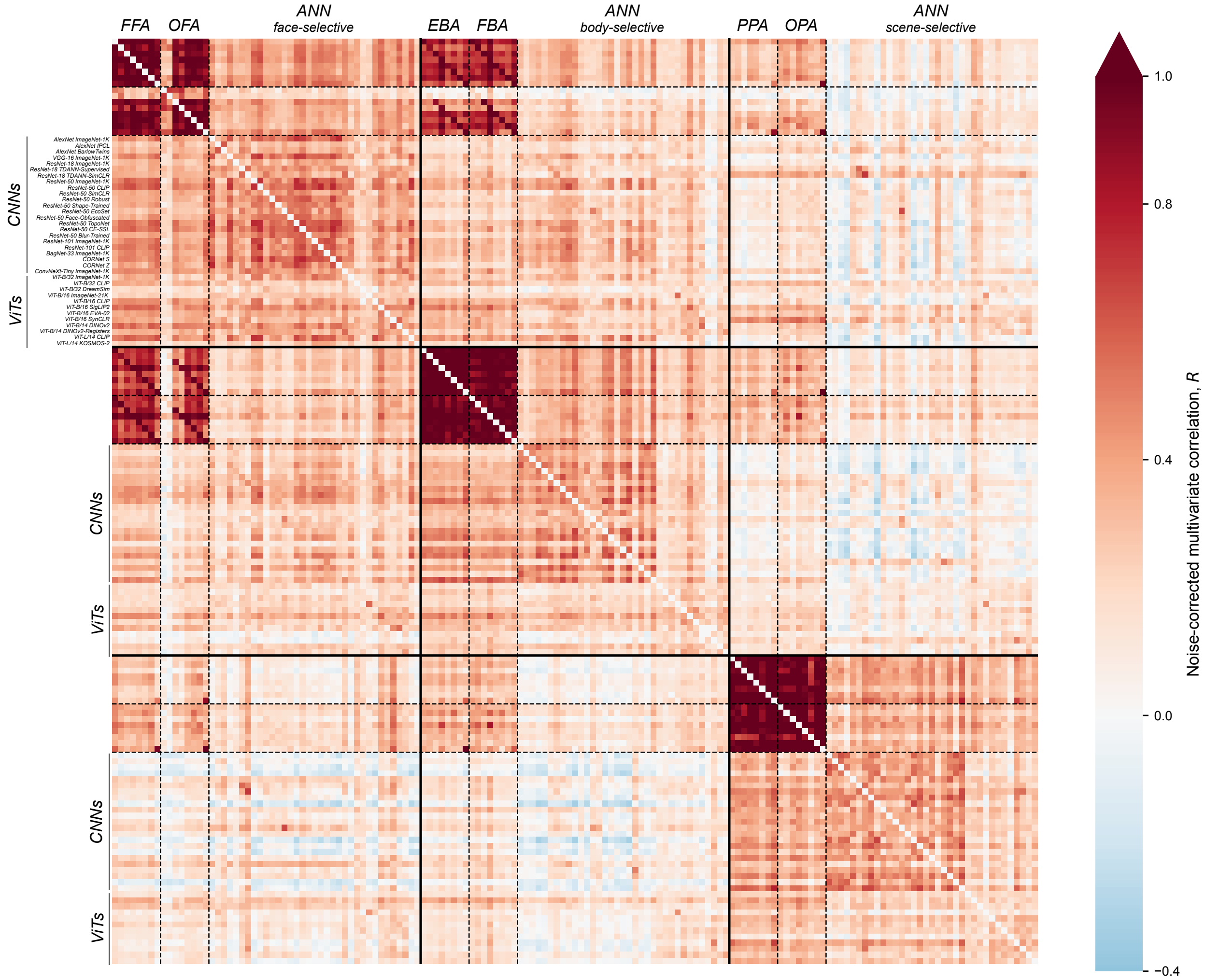

Figure S4 Noise-corrected multivariate correlations across all subjects and trained models, for all category-selective regions. Noise-corrected multivariate correlations between all pairs of subjects and models, across all selectivities and an extended set of category-selective regions. ANN representative layers correspond to canonical category-selective regions, i.e., FFA, EBA, and PPA. Subjects are arranged numerically (1-8), and models are grouped based on their architectures, with model names annotated.

bioRxiv preprint doi: https://doi.org/10.64898/2026.05.29.728609; this version posted June 2, 2026. The copyright holder for this preprint (which was not certified by peer review) is the author/funder, who has granted bioRxiv a license to display the preprint in perpetuity. It is made available under a CC-BY 4.0 International license.

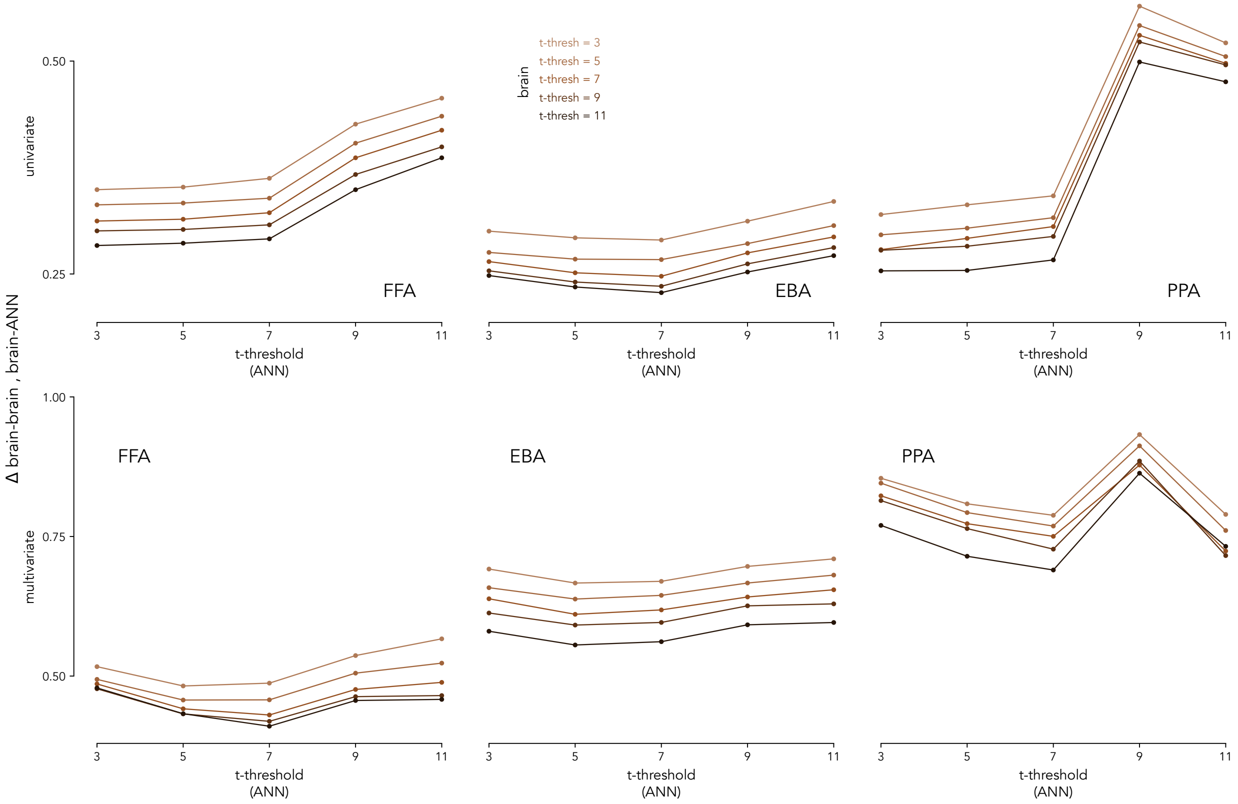

Figure S5 Univariate and multivariate correlation gaps between brains and best-performing model across combinations of brain and ANN selectivity thresholds. Line plots show the noise-corrected correlation gap between the best model (ViT-B/16 SigLIP2) and brains across category-selective regions and comparisons. For each region, the x-axis shows the ANN t-threshold, whereas the y-axis shows the gap in noise-corrected correlations. The gap is the difference between a subject's median correlation with other subjects and correlation with the model (median across subjects). Lines of different shades show the gap for sets of voxels corresponding to different values of Brain's t-threshold, with darker shades corresponding to higher t-threshold. The top row shows the correlation estimated using univariate comparisons, and the bottom row shows the correlations estimated using multivariate comparisons.

bioRxiv preprint doi: https://doi.org/10.64898/2026.05.29.728609; this version posted June 2, 2026. The copyright holder for this preprint (which was not certified by peer review) is the author/funder, who has granted bioRxiv a license to display the preprint in perpetuity. It is made available under a CC-BY 4.0 International license.

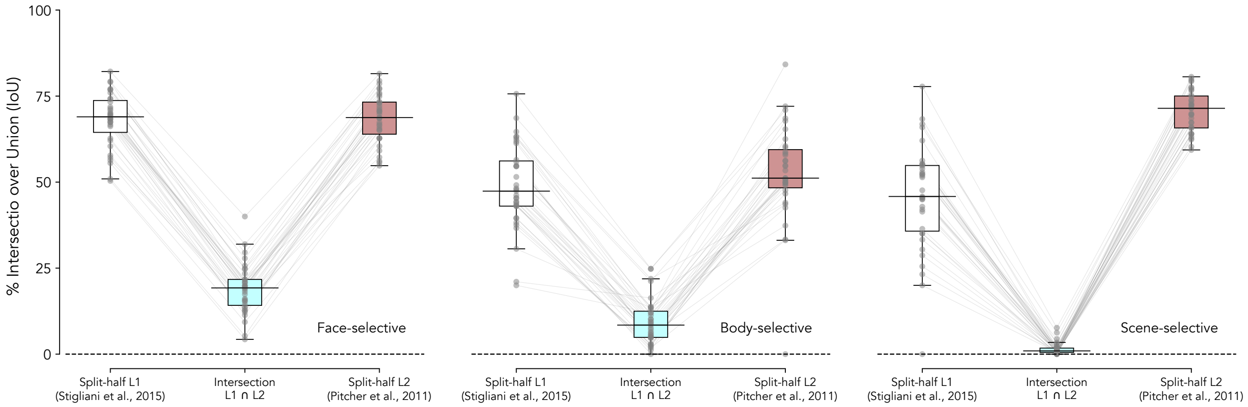

Figure S6 Intersection of unit sets identified within and across different localizers. Boxplots show the percentage overlap (Intersection over Union; IoU) between unit subsets identified using two different functional localizers (intersection $L1 \cap L2$; same as Fig. 4), compared with the within-localizer split-half consistency for each localizer ($L1$ and $L2$). Each point represents one ANN model, and lines connect values from the same model. Across all selectivity domains (face, body, and scene), the overlap between units identified by different localizers is substantially lower than the within-localizer split-half overlap, indicating that the specific stimulus set used for localization strongly influences which ANN units are identified as category-selective.

bioRxiv preprint doi: https://doi.org/10.64898/2026.05.29.728609; this version posted June 2, 2026. The copyright holder for this preprint (which was not certified by peer review) is the author/funder, who has granted bioRxiv a license to display the preprint in perpetuity. It is made available under a CC-BY 4.0 International license.

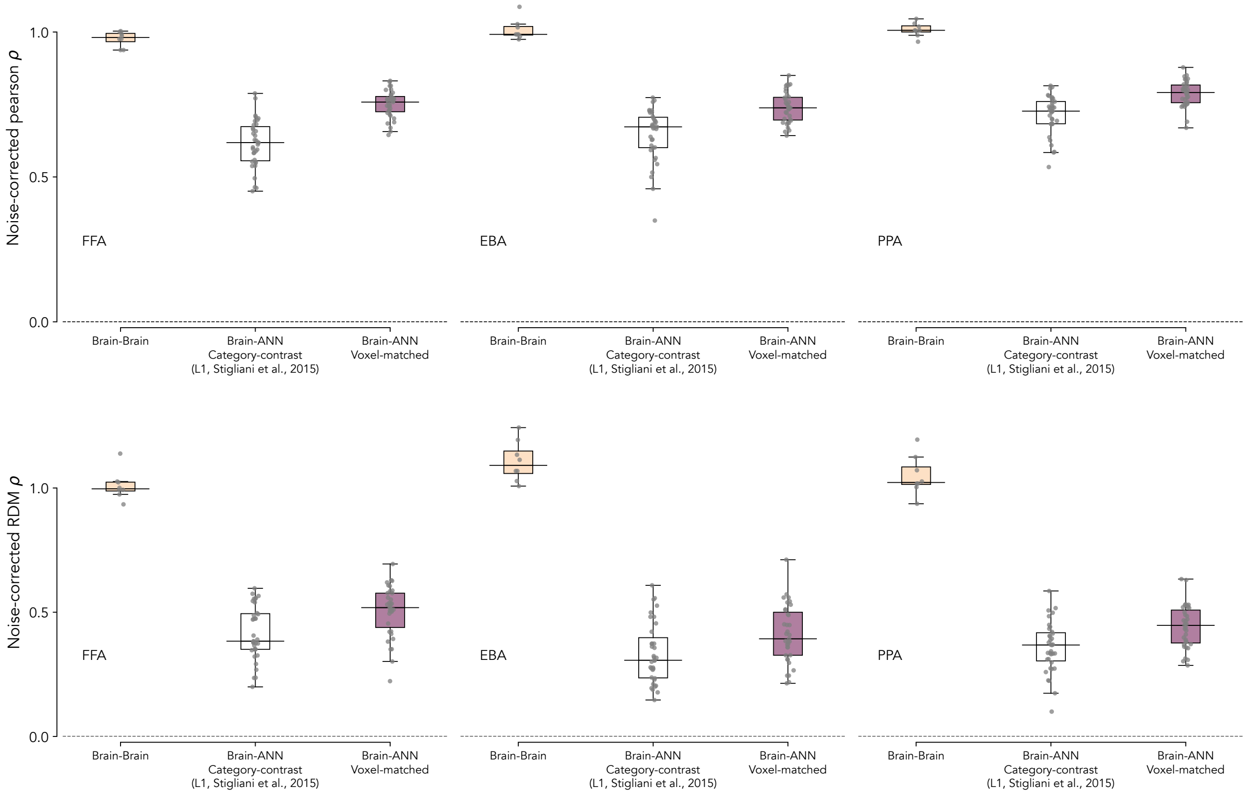

Figure S7 Noise-corrected univariate and multivariate correlations for voxel-matched ANN unit subsets. Descriptive boxplots illustrating the category-selective region (x-axis) v/s. noise-corrected correlation (y-axis) when evaluated on the NSD dataset. For each region, the first boxplot depicts the correlation of responses between human brains (each point indicates one subject's median correlation with all other subjects); the second and third boxplots depict the correlation of responses between the ANN models and human brains for category-selective units identified using functional localizer 1 (same as Fig. 2b) and through voxel-tuning matching procedure, respectively (each point indicates one ANN model's median correlation across all subjects). The ANN representative layer corresponding to fROI is the same as selected previously using functional localizer 1. The top row shows the correlation estimated using univariate comparisons, and the bottom row shows the correlations estimated using multivariate comparisons.

bioRxiv preprint doi: https://doi.org/10.64898/2026.05.29.728609; this version posted June 2, 2026. The copyright holder for this preprint (which was not certified by peer review) is the author/funder, who has granted bioRxiv a license to display the preprint in perpetuity. It is made available under a CC-BY 4.0 International license.

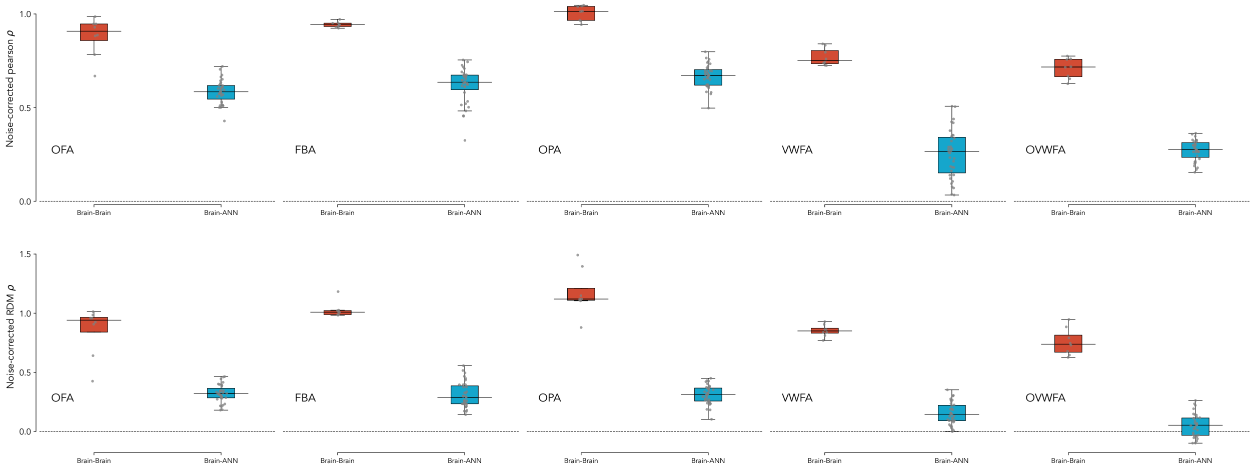

Figure S8 Noise-corrected univariate and multivariate correlations across an extended set of category-selective regions. Descriptive boxplots illustrating the category-selective region (x-axis) v/s. noise-corrected correlation (y-axis) when evaluated on the NSD dataset. For each region, the left boxplot depicts the correlation of responses between human brains (each point indicates one subject's median correlation with all other subjects); the right boxplot depicts the correlation of responses between the ANN models and human brains (each point indicates one ANN model's median correlation across all subjects). Here, the category-selective units were localized using functional localizer 1 (same as Fig. 2), and the layer was selected independently for each fROI. The top row shows the correlation estimated using univariate comparisons, and the bottom row shows the correlations estimated using multivariate comparisons.

bioRxiv preprint doi: https://doi.org/10.64898/2026.05.29.728609; this version posted June 2, 2026. The copyright holder for this preprint (which was not certified by peer review) is the author/funder, who has granted bioRxiv a license to display the preprint in perpetuity. It is made available under a CC-BY 4.0 International license.

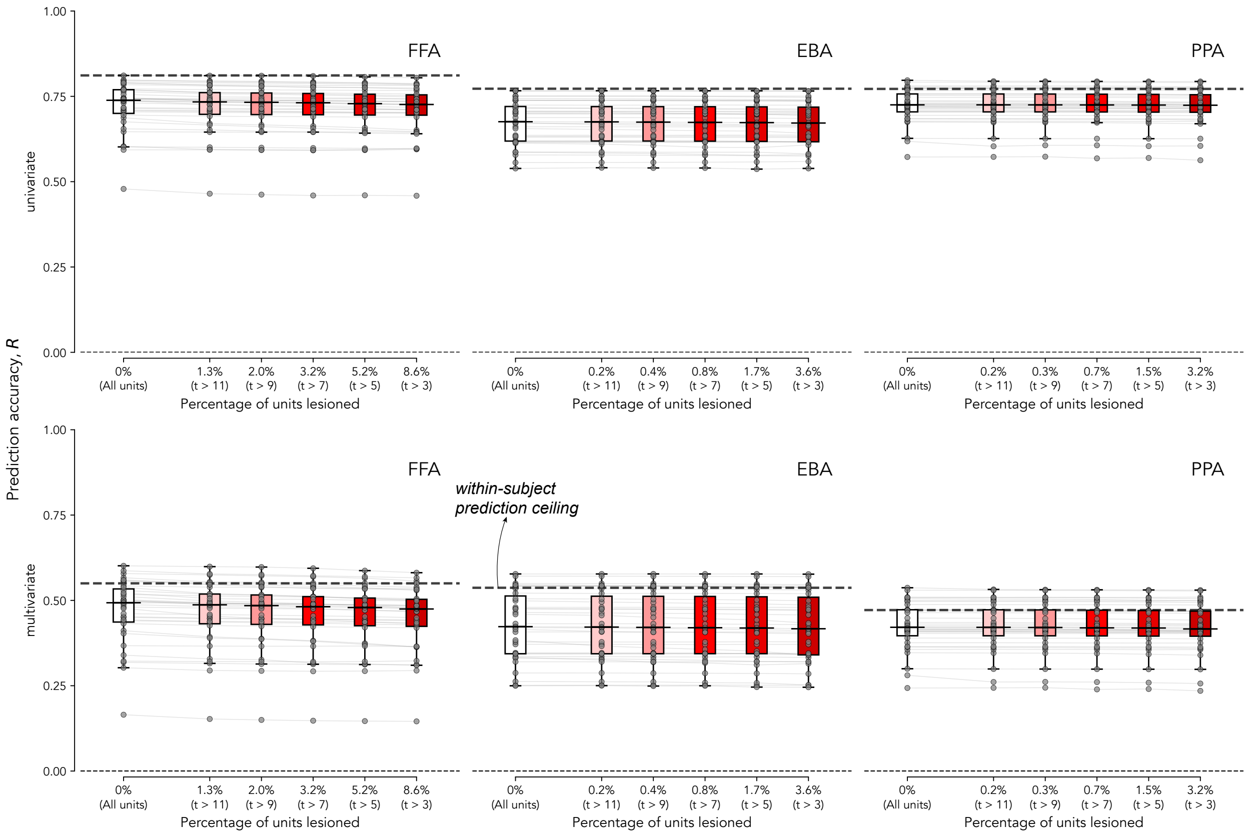

Figure S9 Prediction accuracy of voxel-wise encoding models when category-selective units are lesioned, across selectivity thresholds. Correlation between observed and predicted responses from voxel-wise encoding models (using ridge mapping) on held-out NSD images. For each boxplot, the x-axis indicates the percentage of category-selective units lesioned (category corresponding to the fROI's preferred category), and the y-axis indicates the correlation. The x-axis labels also annotate the corresponding selectivity threshold for the category-selective unit identification. Each box depicts the correlation of the encoding model (each point indicating one ANN model's median correlation across the subjects). The first box indicates the baseline using all units in the layer (previously chosen representative layer corresponding to the fROI). The black dashed line shows the median within-subject ceiling, i.e., spearman-brown corrected split-half correlation between the subject's responses during the three repetitions of the same stimuli. The two rows indicate univariate and multivariate comparisons, respectively. The three columns indicate the category-selective region, i.e., FFA, EBA, and PPA, respectively.

bioRxiv preprint doi: https://doi.org/10.64898/2026.05.29.728609; this version posted June 2, 2026. The copyright holder for this preprint (which was not certified by peer review) is the author/funder, who has granted bioRxiv a license to display the preprint in perpetuity. It is made available under a CC-BY 4.0 International license.

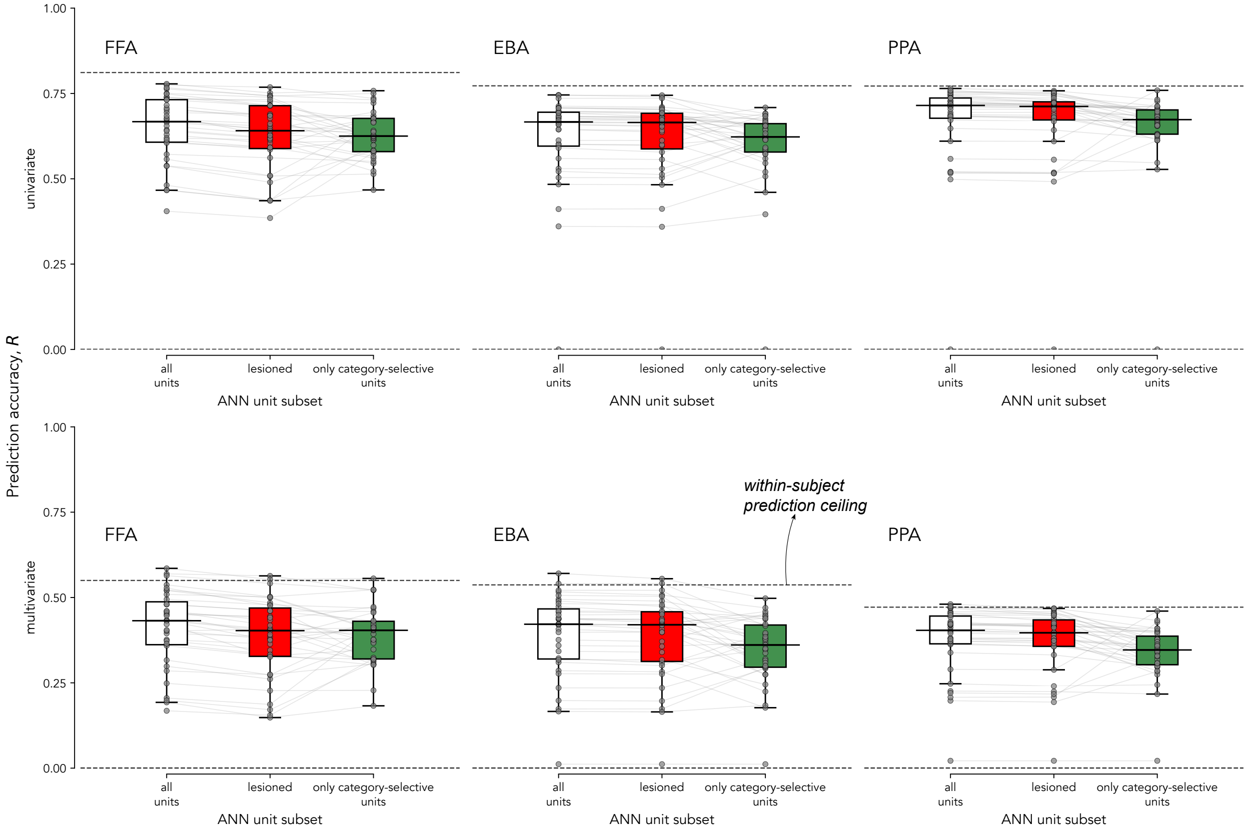

Figure S10 Prediction accuracy of voxel-wise encoding models when category-selective units are lesioned, using sparse-positive mapping. Correlation between observed and predicted responses from voxel-wise encoding models (using sparse-positive mapping) on held-out NSD images. For each boxplot, the x-axis indicates the unit set considered, and the y-axis indicates the correlation. The first box depicts the correlation of the encoding model using all the units within the ANN layer (each point indicating one ANN model's median correlation across the subjects); similarly, the second and third boxes correspond to encoding models when the category-selective units are lesioned, and when only category-selective units are considered. The black dashed line shows the median within-subject ceiling, i.e., spearman-brown corrected split-half correlation between the subject's responses during the three repetitions of the same stimuli. The two rows indicate univariate and multivariate comparisons, respectively. The three columns indicate the category-selective region, i.e., FFA, EBA, and PPA, respectively.

bioRxiv preprint doi: https://doi.org/10.64898/2026.05.29.728609; this version posted June 2, 2026. The copyright holder for this preprint (which was not certified by peer review) is the author/funder, who has granted bioRxiv a license to display the preprint in perpetuity. It is made available under a CC-BY 4.0 International license.

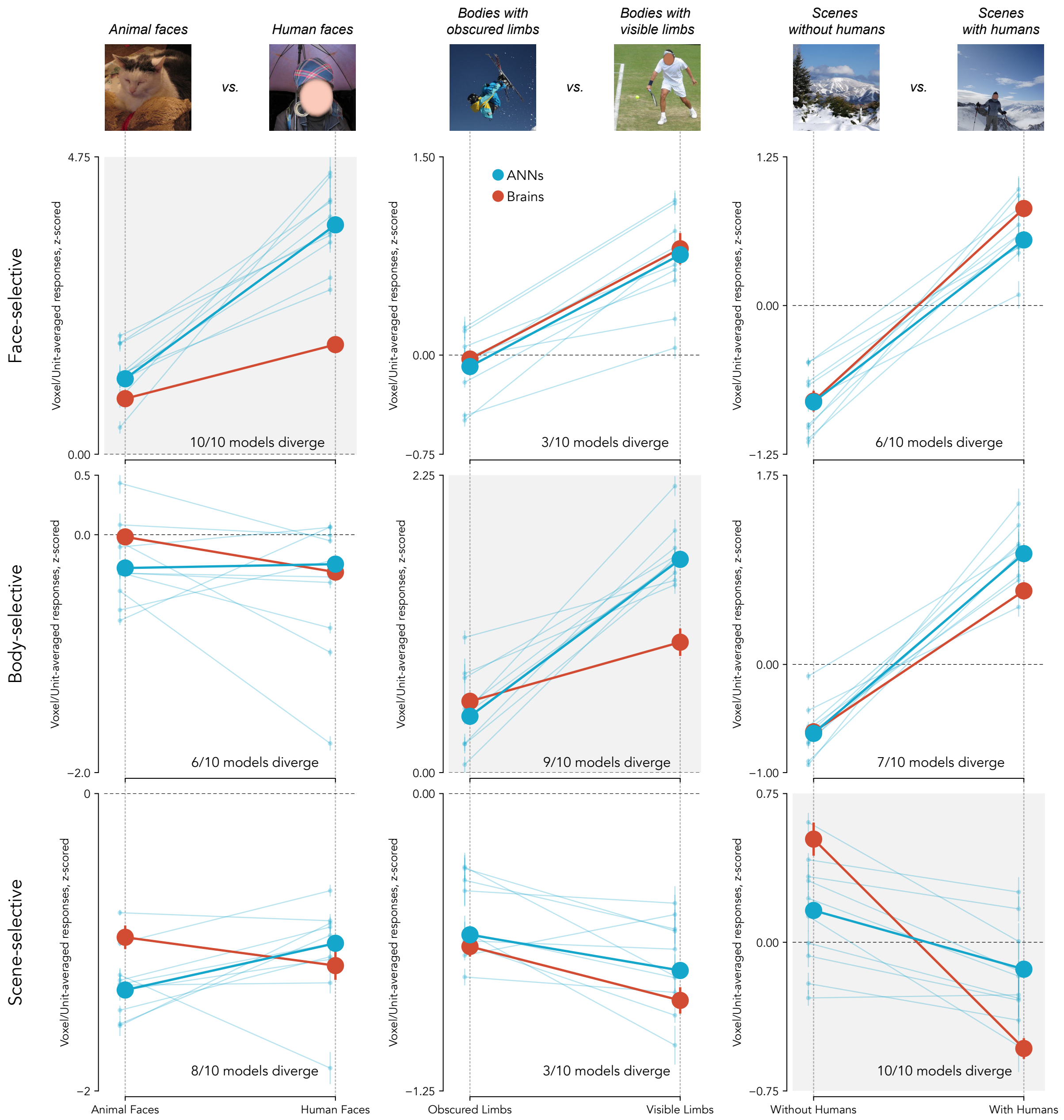

Figure S11 Brain and ANN responses for chosen stimulus groups, across all selectivities. Hypothesis validation of the candidate stimulus-level factors when evaluated on independent, subject-specific held-out images, held-out subjects, and held-out top-10 most brain-aligned models according to univariate tests. The top panel shows example images from the stimulus groups that were hypothesized to produce systematic divergence between ANN and brain responses from three domains of category selectivity: face-selective (animal faces vs. human faces), body-selective (bodies with obscured limbs vs. visible limbs), and scene-selective (scenes without humans vs. scenes with humans) (same as Fig. 6). A total of 350 stimuli were used for evaluation (25 per group per subject). The line plots show a descriptive visualization of ANN–brain divergence for each stimulus group. Three columns show the stimulus groups, and the three rows show the evaluated selectivities (and the corresponding fROI, i.e., FFA, EBA, and PPA, respectively). The shaded plot depicts hypotheses generated and evaluated within selectivity (here, the plots are the same as Fig. 6). For each plot, the x-axis indicates stimulus group, and the y-axis shows unit-averaged (ANN) or voxel-averaged (brain) z-scored responses. In all plots, the thick red lines denote the mean response across subjects (points indicate mean ± SEM), thick blue lines denote the median response across the models, and faint blue lines show individual model responses. The annotated text shows the number of models (out of the top-10) that diverge, i.e., have a significant group × system interaction effect using the linear mixed-effects model as before.

bioRxiv preprint doi: https://doi.org/10.64898/2026.05.29.728609; this version posted June 2, 2026. The copyright holder for this preprint (which was not certified by peer review) is the author/funder, who has granted bioRxiv a license to display the preprint in perpetuity. It is made available under a CC-BY 4.0 International license.

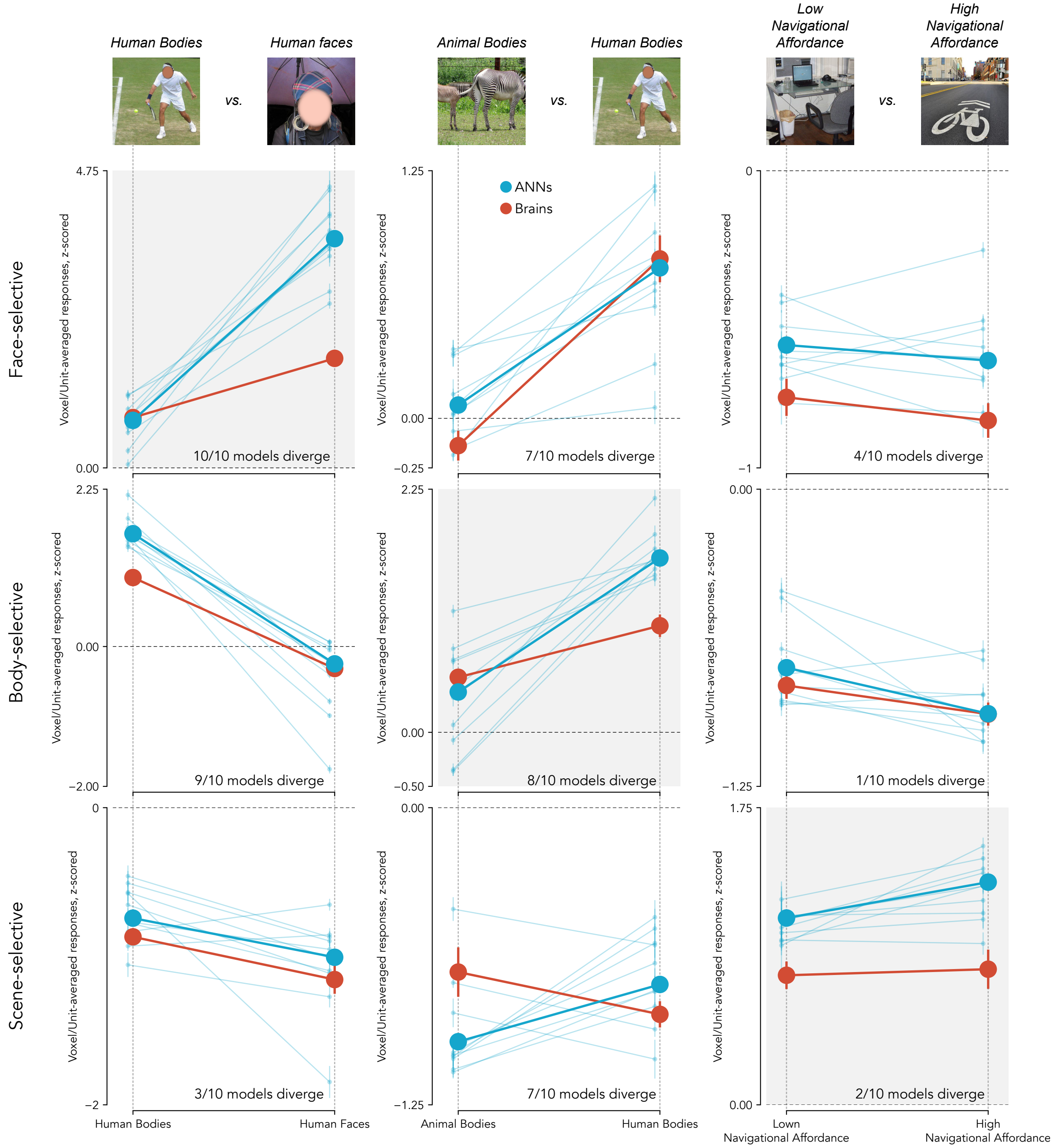

Figure S12 Brain and ANN responses for extended stimulus groups, across all selectivities. Hypothesis validation of the candidate stimulus-level factors when evaluated on independent, subject-specific held-out images, held-out subjects, and held-out top-10 most brain-aligned models according to univariate tests. The top panel shows example images from the stimulus groups that were hypothesized to produce systematic divergence between ANN and brain responses from three domains of category selectivity: face-selective (human bodies vs. faces), body-selective (animal vs. human bodies), and scene-selective (low vs. high navigational affordances) (human faces, and human bodies groups are the same as Fig. 6 and Fig. S11). A total of 350 stimuli were used for evaluation (25 per group per subject). The line plots show a descriptive visualization of ANN–brain divergence for each stimulus group. Three columns show the stimulus groups, and the three rows show the evaluated selectivities (and the corresponding fROI, i.e., FFA, EBA, and PPA, respectively). The shaded plot depicts hypotheses generated and evaluated within selectivity. For each plot, the x-axis indicates stimulus group, and the y-axis shows unit-averaged (ANN) or voxel-averaged (brain) z-scored responses. In all plots, the thick red lines denote the mean response across subjects (points indicate mean ± SEM), thick blue lines denote the median response across the models, and faint blue lines show individual model responses. The annotated text shows the number of models (out of the top-10) that diverge, i.e., have a significant group × system interaction effect using the linear mixed-effects model as before.

bioRxiv preprint doi: https://doi.org/10.64898/2026.05.29.728609; this version posted June 2, 2026. The copyright holder for this preprint (which was not certified by peer review) is the author/funder, who has granted bioRxiv a license to display the preprint in perpetuity. It is made available under a CC-BY 4.0 International license.

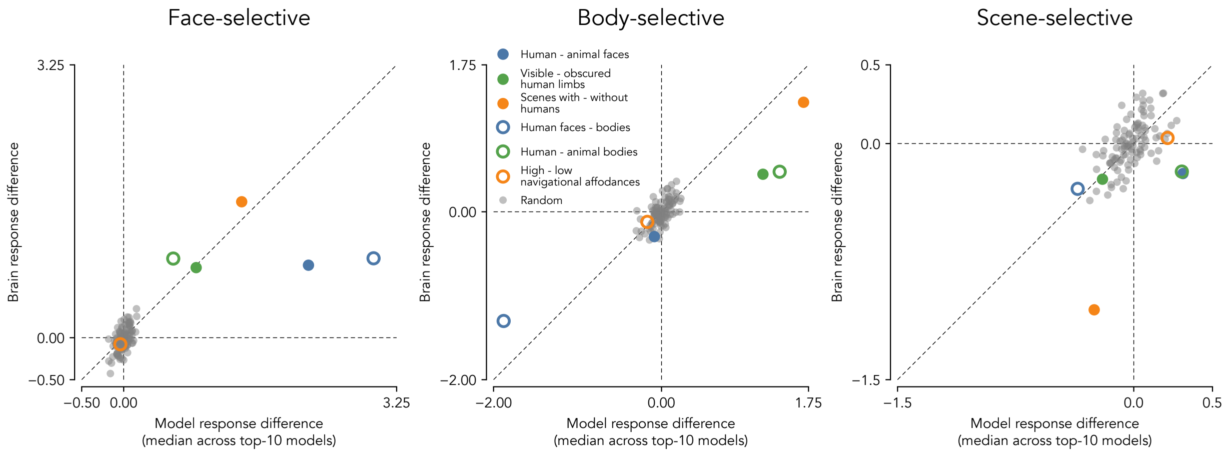

Figure S13 Brain and ANN responses for all stimulus groups, including random, across all selectivities. Each panel shows the difference in responses between the two stimulus groups for the brain (y-axis) and ANN models (x-axis). Brain response differences correspond to voxel-averaged responses in the corresponding category-selective region (FFA, EBA, or PPA) (median of within-subject difference, subjects 2-7), and model response differences correspond to unit-averaged responses from the top-10 most brain-aligned models (median of within-subject difference for each model, then median across models). Each colored point represents a stimulus contrast hypothesized to produce divergent responses between ANNs and brains across three domains of category selectivity: face-selective (animal faces vs. human faces), body-selective (bodies with obscured limbs vs. visible limbs), and scene-selective (scenes without humans vs. scenes with humans) (same as Fig. 6, S11). Additional contrasts tested across domains: face-selective (human bodies vs. faces), body-selective (animal vs. human bodies), and scene-selective (low vs. high navigational affordances) (same as Fig. S12). Gray points indicate control analyses using randomly sampled stimulus groups (100 bootstrap samples; each sample contains 350 stimuli total, 25 per group per subject) drawn from the same held-out subject-specific image sets (excluding the stimuli selected in previous hypotheses). These random contrasts cluster around the origin and do not show systematic divergence. The dashed diagonal line indicates equality between model and brain response differences. Points that lie far from this line indicate stimulus contrasts where ANN and brain responses diverge. Across selectivities, the hypothesized stimulus groups consistently show larger response differences in ANNs than in the corresponding brain regions, whereas random stimulus groups show no such divergence.

bioRxiv preprint doi: https://doi.org/10.64898/2026.05.29.728609; this version posted June 2, 2026. The copyright holder for this preprint (which was not certified by peer review) is the author/funder, who has granted bioRxiv a license to display the preprint in perpetuity. It is made available under a CC-BY 4.0 International license.

Table 1 Summary of all the pretrained models evaluated

#	Model name	Optimization Type	Architecture	Training Objective	Citation
1	ViT-B/16 SigLIP2	Task-optimized	Transformer	Language-aligned	[113]
2	ResNet-50 CLIP	Task-optimized	CNN	Language-aligned	[109]
3	ConvNeXt-Tiny ImageNet-1K	Task-optimized	CNN	Supervised	[114]
4	ResNet-101 CLIP	Task-optimized	CNN	Language-aligned	[109]
5	ViT-B/14 DINOv2-Registers	Task-optimized	Transformer	Self-supervised	[115]
6	ResNet-50 SimCLR	Task-optimized	CNN	Self-supervised	[116]
7	ResNet-50 TopoNet ($\tau = 30$)	Task-optimized	CNN	Supervised	[29]
8	CORNet S	Task-optimized	CNN	Supervised	[96]
9	ResNet-101 ImageNet-1K	Task-optimized	CNN	Supervised	[117]
10	ViT-B/14 DINOv2	Task-optimized	Transformer	Self-supervised	[118]
11	ResNet-50 CE-SSL (BarlowTwins)	Task-optimized	CNN	Self-supervised	[119]
12	ResNet-18 ImageNet-1K	Task-optimized	CNN	Supervised	[117]
13	ResNet-50 ImageNet-1K	Task-optimized	CNN	Supervised	[117]
14	ResNet-50 Face-Obfuscated	Task-optimized	CNN	Supervised	[120]
15	ViT-B/16 EVA-02	Task-optimized	Transformer	Language-aligned	[121]
16	ResNet-50 EcoSet	Task-optimized	CNN	Supervised	[92]
17	ViT-L/14 KOSMOS-2	Task-optimized	Transformer	Language-Aligned	[122]
18	ViT-B/16 SynCLR	Task-optimized	Transformer	Self-supervised	[123]
19	ResNet-18 TDANN-SimCLR	Task-optimized	CNN	Self-supervised	[30]
20	VGG-16 ImageNet-1K	Task-optimized	CNN	Supervised	[124]
21	ViT-B/32 CLIP	Task-optimized	Transformer	Language-aligned	[109]
22	ViT-B/16 CLIP	Task-optimized	Transformer	Language-aligned	[109]
23	ViT-L/14 CLIP	Task-optimized	Transformer	Language-Aligned	[109]

Continued on next page

bioRxiv preprint doi: https://doi.org/10.64898/2026.05.29.728609; this version posted June 2, 2026. The copyright holder for this preprint (which was not certified by peer review) is the author/funder, who has granted bioRxiv a license to display the preprint in perpetuity. It is made available under a CC-BY 4.0 International license.

Table 1 – continued from previous page

#	Model name	Optimization Type	Architecture	Training Objective	Citation
24	ResNet-50 Robust (L2 $\epsilon = 3$)	Task-optimized	CNN	Supervised	[125]
25	ViT-B/32 DreamSim (Open CLIP)	Task-optimized	Transformer	Language-Aligned	[126]
26	ResNet-50 Blur-Trained (Strong)	Task-optimized	CNN	Supervised	[88]
27	ResNet-50 Shape-Trained	Task-optimized	CNN	Supervised	[127]
28	BagNet-33 ImageNet-1K	Task-optimized	CNN	Supervised	[128]
29	ViT-B/32 ImageNet-1K	Task-optimized	Transformer	Supervised	[129]
30	AlexNet ImageNet-1K	Task-optimized	CNN	Supervised	[130]
31	AlexNet BarlowTwins	Task-optimized	CNN	Self-supervised	[24, 131]
32	ViT-B/16 ImageNet-21K	Task-optimized	Transformer	Supervised	[132]
33	ResNet-18 TDANN-Supervised	Task-optimized	CNN	Supervised	[30]
34	CORNet Z	Task-optimized	CNN	Supervised	[96]
35	AlexNet IPCL (alexnetgn_ipcl_ref01)	Task-optimized	CNN	Self-supervised	[110]

Table 2 Summary of all the untrained model architectures evaluated

#	Model name	Optimization Type	Architecture	Training Objective	Citation
1	AlexNet	Untrained	CNN	-	[130]
2	ResNet-18	Untrained	CNN	-	[117]
3	ResNet-50	Untrained	CNN	-	[117]
4	ViT-B/32	Untrained	Transformer	-	[129]
